# Dihydroartemisinin-induced unfolded protein response feedback attenuates ferroptosis via PERK/ATF4/HSPA5 pathway in glioma cells

**DOI:** 10.1186/s13046-019-1413-7

**Published:** 2019-09-13

**Authors:** Yibing Chen, Yanjun Mi, Xiaofei Zhang, Qian Ma, Yucen Song, Liwei Zhang, Dandan Wang, Jinliang Xing, Benxin Hou, Haolong Li, Huan Jin, Wei Du, Zhengzhi Zou

**Affiliations:** 1 Genetic and Prenatal Diagnosis Center, Department of Gynecology and Obstetrics, First Affiliated Hospital, Zhengzhou University, Zhengzhou, 450052 China; 2 Department of Medical Oncology, Xiamen Cancer Hospital, First Affiliated Hospital of Xiamen University, Xiamen, 361003 China; 3 Department of Medical Oncology, First Affiliated Hospital, Zhengzhou University, Zhengzhou, 450052 China; 4 Department of Neurosurgery, First Affiliated Hospital, Zhengzhou University, Zhengzhou, 450052 China; 5 Shandong Medicinal Biotechnology Centre, Key Laboratory for Rare & Uncommon Diseases of Shandong Province, Back and Neck Pain Hospital of Shandong Academy of Medical Sciences, Shandong Academy of Medical Sciences, Jinan, 250062 China; 6 State Key Laboratory of Cancer Biology & Experimental Teaching Center of Basic Medicine, Fourth Military Medical University, Xi’an, 710032 China; 7 Department of General Surgery, Hainan Province Nongken Sanya Hospital, Sanya, 572000 China; 8 MOE Key Laboratory of Laser Life Science and Institute of Laser Life Science, College of Biophotonics, South China Normal University, Guangzhou, 510631 Guangdong China

**Keywords:** Dihydroartemisinin, Ferroptosis, GPX4, Endoplasmic reticulum stress, HSPA5, Glioma

## Abstract

**Background:**

Dihydroartemisinin (DHA) has been shown to exert anticancer activity through iron-dependent reactive oxygen species (ROS) generation, which is similar to ferroptosis, a novel form of cell death. However, whether DHA causes ferroptosis in glioma cells and the potential regulatory mechanisms remain unclear.

**Methods:**

Effects of DHA on the proliferation, cell death, ROS and lipid ROS generation as well as reduced gluthione consumption were assessed in glioma cells with or without ferroptosis inhibitor. The biological mechanisms by which glioma cells attenuate the pro-ferroptotic effects of DHA were assessed using molecular methods.

**Results:**

DHA induced ferroptosis in glioma cells, as characterized by iron-dependent cell death accompanied with ROS generation and lipid peroxidation. However, DHA treatment simultaneously activated a feedback pathway of ferroptosis by increasing the expression of heat shock protein family A (Hsp70) member 5 (HSPA5). Mechanistically, DHA caused endoplasmic reticulum (ER) stress in glioma cells, which resulted in the induction of HSPA5 expression by protein kinase R-like ER kinase (PERK)-upregulated activating transcription factor 4 (ATF4). Subsequent HSPA5 upregulation increased the expression and activity of glutathione peroxidase 4 (GPX4), which neutralized DHA-induced lipid peroxidation and thus protected glioma cells from ferroptosis. Inhibition of the PERK-ATF4-HSPA5-GPX4 pathway using siRNA or small molecules increased DHA sensitivity of glioma cells by increasing ferroptosis both in vitro and in vivo.

**Conclusions:**

Collectively, these data suggested that ferroptosis might be a novel anticancer mechanism of DHA in glioma and HSPA5 may serve as a negative regulator of DHA-induced ferroptosis. Therefore, inhibiting the negative feedback pathway would be a promising therapeutic strategy to strengthen the anti-glioma activity of DHA.

## Background

Glioma is the most common primary brain tumor in both children and adults, and accounting for 7% of cancer-related death before the age of 70 years [1]. Despite significant improvement in treatment for glioma patients, the survival remains not optimistic, particularly for those with glioblastoma. Patients with newly diagnosed glioblastoma exhibit one-year survival rate of 35.7% and five-year survival rate of 4.7%, with generally poor responses to most of current available therapeutic modalities [2]. Therefore, there is an urgent need to develop novel effective therapies with low toxicity for the treatment of glioma based on molecular studies.

Regulated cell death modalities are emerging as important players in physiological conditions and diseases, in which caspase-dependent apoptosis is the mostly studied regulated cell death form [3]. In recent years, growing evidence has indicated that regulated cell death mechanisms beyond apoptosis also play important roles in the molecular processes controlling the demise of cells. Ferroptosis is a recently recognized form of regulated cell death characterized by the accumulation of lipid peroxidation products and lethal reactive oxygen species (ROS) derived from iron metabolism, which is genetically, biochemically and morphologically distinct from other forms of cell death [4]. An increasing number of studies have implicated that ferroptosis is involved in various human diseases, including neurodegenerative diseases, ischemia-reperfusion injury, and kidney degeneration [5]. In addition, a number of cancer cells have been proven to be susceptive to ferroptosis inducers. Moreover, ferroptosis inducers such as erastin can improve the efficacy of chemotherapeutic reagents, including temozolomide, cisplatin, ara-C and doxorubicin [6]. Therefore, inducing and enhancing ferroptosis may be a promising strategy for cancer therapy.

Artemisinin (ART), the active principle of the Chinese medicinal herb *Artemisia annua L*, and its derivatives have been widely used to treat schistocidal malaria infection with high efficiency and low side effects [7]. Dihydroartemisinin (DHA), a main active derivative of ART, has been demonstrated to exert preferentially cytotoxic effects toward human malignancies from various tissues, including breast, lung carcinoma, colorectal cancers as well as lymphoma [8–11]. Recent studies have shown that the anti-cancer activity of ART derivatives including DHA highly relies on the iron-mediated cleavage of endoperoxide bridge and subsequent ROS generation [10]. Since most cancer cells have higher iron uptake than normal cells, DHA may exert selective toxicity toward cancer cells [12, 13]. Moreover, Ooko et al. have shown that treatment of ART derivatives caused significant changes in the expression of iron-related genes in 60 National Cancer Institute cell lines, indicating the ferroptosis-inducing activity of ART derivatives [14]. Indeed, Lin et al. have preliminarily revealed that DHA induces ferroptosis in head and neck carcinoma cells [15]. However, whether DHA causes ferroptosis in other types of cancer including glioma remains unclear.

The sensitivity to ferroptosis is tightly linked to numerous biological processes, including metabolisms of iron, membrane lipids and amino acid and the redox regulation system [5]. Recent studies have demonstrated that glutathione peroxidase 4 (GPX4), heat shock protein family A (Hsp70) member 5 (HSPA5), heat shock protein beta-1 (HSPB1), nuclear factor erythroid 2-related factor 2 (NRF2) and α6β4 integrin function as negative regulators of ferroptosis by limiting ROS production or reducing cellular iron uptake [16–20], whereas reduced β-nicotinamide adenine dinucleotide phosphate (NADPH) oxidase, heme oxygenase-1 and p53 act as positive regulators of ferroptosis [4, 21, 22]. However, the roles of these molecular players in ferroptosis appear to be drug- or cell type-specific, and thus regulatory mechanisms of ferroptosis induced by other reagents including DHA are still unknown.

Endoplasmic reticulum (ER) stress in solid tumors results from a dysregulation of protein synthesis, folding, secretion, or posttranslational modification, which can be triggered by environmental stimuli such as nutrient deprivation, hypoxia, chronic viral infection, oxidative stress and anticancer drugs [23]. To cope with ER stress, cancer cells initiate an evolutionarily conserved signaling process known as the unfolded protein response (UPR), which is coordinated by three ER transmembrane-bound sensors: inositol-requiring transmembrane kinase/endoribonuclease 1α (IRE1α), activating transcription factor 6 (ATF6) and protein kinase R-like ER kinase (PERK). HSPA5, also termed GRP78 or BiP, is a molecular chaperone located in the ER and acts as a master regulator of UPR [24]. A number of studies have demonstrated that HSPA5 promotes cell survival and drug resistance under ER stress conditions [25]. Recently, Zhu et al. have reported that HSPA5 promotes ferroptosis resistance in pancreatic ductal adenocarcinoma cells [17]. However, whether HSPA5 regulates ferroptosis in glioma cells remains unclear. In this study, we provided the first evidence that DHA induced ferroptosis in glioma cells. Furthermore, we found that DHA also induced the expression of HSPA5 via PERK-ATF4 pathway, which attenuated the sensitivity to ferroptosis through GPX4. Moreover, inhibition of HSPA5 or PERK showed a synergistic anticancer effect with DHA in glioma. These data provided a novel preclinical evidence for the combination of DHA and HSPA5 inhibition in the treatment of glioma.

## Materials and methods

### Antibodies and reagents

Antibodies against HSPA5 (ab21685), CHOP (ab11419), ATF4 (ab184909), GPX4 (ab125066) and xCT (ab60171) were purchased form abcam (Cambridge, MA, USA), ATF6 (#65880), PERK (#5683), and anti-HA tag (#3724 s) antibodies from Cell Signaling Technology (Danvers, MA, USA) and anti-β-actin antibody from Santa Cruz Biotechnology (Dallas, TX, USA). DHA (D7439), ferrostatin-1 (Fer-1, SML0583), liproxstatin-1 (Lip-1, SML1414), GSK2606414 (PERK Inhibitor I, PERKi, 516,535), epigallocatechine gallate (EGCG, 324880) and deferoxamine (DFO, 252750) were supplied by Merck KGaA (Darmstadt, Germany).

### Patient sample and cell culture

Histopathologically-confirmed malignant glioma samples were obtained from patients undergoing surgical treatment at the First Affiliated Hospital of Zhengzhou University. Within 1–3 h after surgical removal, tumors were washed in PBS and enzymatically dissociated into single cells. Patient-derived glioma cells were maintained in astrocyte basal medium (Lonza), supplemented with 10% fetal bovine serum (Carlsbad, CA, USA), L-glutamate, ascorbic acid, rhEGF, insulin, and gentamicin sulphate/amphotericin B according to the manufacturer’s recommendations. This study was approved by the Ethical Committee of Zhengzhou University, and written informed consent was obtained from all participants. All study procedures were carried out in accordance with the ethical standards of the Helsinki Declaration.

Glioma cell lines U251 and U373 and mouse hippocampal neuronal cell line HT22 were purchased from the American Type Culture Collection (Manassas, VA, USA). U251 and U373 cell lines cultured in DMEM medium supplemented with 10% Gibco fetal bovine serum. All cells were kept at 37 °C in a 5% CO_2_ humidified incubator. All cells were *Mycoplasma* free and authenticated by short tandem repeat DNA profiling analysis.

### Cell viability assay

Cell viability was assessed using a Promega Cell Titer 96 Aqueous One Solution (G3580, Madison, WI, USA) as previously described [26]. Briefly, cells were seeded in 96-well plates (500 per well) in 100 μl DEME medium for five replicates. On the day of assay, 20 μl Cell Titer 96 Aqueous One Solution were added into the medium and incubated at 37 °C for 4 h. The absorbance at 490 nm (OD490) was measured on a microplate spectrophotometer. Values are expressed ratio to the vehicle-treated control.

### Colony formation assay

Log-phase growing cells were seeded into 6-well plates at a density of 800 cells/ well and cultured for 14 days. Then cells were washed, fixed and stained with 0.5% crystal violet. Colonies that contained > 50 stained cells were classed as clones. Colony-forming efficiency was calculated as a ratio of the number of colonies formed to the number of cells seeded and calibrated to that of untreated cells as previously described [26].

### Cell death analysis

Cell death was analyzed using Annexin-V/7AAD (BD Pharmingen) on a FACS Calibur flow cytometer (BD Biosciences, San Jose, CA, USA). Cells undergoing cell death were analyzed by counting the cells that stained positive for Annexin V-FITC or/and 7-ADD.

### Intracellular ROS analysis

ROS was stained with Dihydroethidium (DHE, Merck KGaA) and detected using flow cytometry according to our previous protocol [27]. Briefly, cells were trypsinized and washed, and then incubated with 1.25 μM DHE for 30 min at 37 °C in dark. Fluorescence was detected on a FACS Calibur™ flow cytometer at the emission wave length of 610 nm.

### Lipid ROS analysis

Lipid ROS was stained with C11-BODIPY 581/591 (D3861, ThermoFisher Scientific, Shanghai, China) and analyzed using flow cytometry as previously described [4]. Briefly, cells were trypsinized, incubated in Hanks Balanced Salt Solution (HBSS) with 2 μM DHE for 10 min at 37 °C in dark and the resuspended in fresh HBSS. Oxidation of the polyunsaturated butadienyl portion of the dye resulted in a shift of the fluorescence emission peak from ~ 590 nm to ~ 510 nm that could be detected on the FACS Calibur™ flow cytometer.

### Malondialdehyde (MDA) determination

To assay lipid peroxidation, the malondialdehyde (MDA) lipid peroxidation microplate assay (Merck KGaA) was used according to manufacturers’ instructions as previously described [20]. In brief, cells were collected by trypsinization, lysed, and reacted with thiobarbituric acid. Fluorescence was measured on a Micro-plate Reader (Bio-Rad, Hercules, CA, USA) using excitation and emission filters of 532 and 590 nm, respectively. Lipid peroxidation levels were normalized to protein concentration.

### Gluthione determination

Intracellular reduced form gluthione (GSH) and its oxidized form (GSSG) were assessed using a GSH/GSSG Ratio Detection Assay Kit II (Abcam) according to the manufacturer’s instructions. Briefly, 50 μl of GSH Assay Mixture (for GSH) or Total GSH Assay Mixture (for total gluthione) solution was added to 50 μl of cell lysate sample in a 96-well plate and then incubated from light for 30 min. The output was measured on a fluorescence microplate reader at Ex/Em = 490/520 nm and concentrations of GSH and total gluthione were calculated using corresponding standard curves. GSSG concentration was calculated as following: GSSG = (total gluthione - GSH)/2.

### Western blot

Cells were lysed in RIPA lysis buffer, and lysates were harvested by centrifugation (12,000 rpm) at 4 °C for 30 min. Western blot was performed according to protocols as previously described [26], using β-actin as the internal control. Briefly, 20 μg of the protein sample was separated by sodium dodecyl sulfate polyacrylamide gel electrophoresis and then transferred to a polyvinylidene fluoride membrane. After blocking nonspecific binding sites for 60 min with 5% non-fat milk, the membrane was incubated with the primary antibody at 4 °C overnight. Membranes were washed three times with tris buffered saline with 1‰ tween-20 and incubated with horseradish peroxidase-conjugated secondary antibody at 37 °C for 1 h. After 3 washes, the bands were detected by an enhanced chemiluminescence system (WBKLS0500, Merck KGaA). Band density was measured using ImageJ software (National Institutes of Health, Bethesda, MD) and standardized to that of β-actin. Antibodies against proteins and dilution multiples are as following: HSPA5 (1:2000), CHOP (1:1000), ATF4 (1,1000), GPX4 (1:2000), HA tag (1:2000) and β-actin (12000).

### RNA extraction and real-time PCR

Total RNA was extracted using TRIzol reagent (15596026, ThermoFisher Scientific) according to the manufacturer’s protocol. Then, 2 μg of RNA was reverse transcribed into first-strand cDNA by M-MLV Reverse Transcriptase (M1705, Promega, Madison, WI, USA) according to the manufacturer’s instructions. Gene-specific amplification was performed in an ABI 7900HT real-time PCR system (Life Technologies, Carlsbad, CA, USA) as previously described [26]. The reaction system contained 0.5 μl cDNA, 7.5 μl 2× SYBR Green PCR master mix (4309155, ThermoFisher Scientific), and 200 nM of the appropriate oligonucleotide primers. All measurements were performed in triplicate, after undergoing the following reaction cycle: preheat at 95 °C for 10 min, 40 cycles of 95 °C for 30 s, and 60 °C for 1 min. The melting curve was measured at 95 °C for 15 s, 60 °C for 15 s, and 72 °C for 15 s. The Ct (threshold cycle) value of each sample was measured during exponential amplification, and was calculated from threshold cycles with the SDS 2.3 software. The relative expression of mRNA was normalized to β-actin (2^-ΔΔCt^ method). Primers were used as following: HSPA5 forward: 5′-CACGGTCTTTGACGCCAAG-3′, HSPA5 reverse: 5′-CCAAATAAGCCTCAGCGGTTT-3′; β-actin forward: 5′-CATGTACGTTGCTATCCAGGC-3′, β-actin reverse: 5′-CTCCTTAATGTCACGCACGAT-3′.

### Gene and siRNA transfection

cDNA of full-length open reading frame of *HSPA5* (NM_005347.4) and *GPX4* (NM_002085.4) was amplified, digested with Xho I and Hind III enzymes and cloned into pLVPT expression vector. GPX4 siRNAs (sc44465) were purchased from Santa Cruz. siRNAs against HSPA5, ATF4 and negative control (NC) were synthesized by Shanghai GenePharma (Shanghai, China). siRNA sequences were designed as following: NC, 5′-AAGGUGGUUGUUUUGUUCACU-3′; HSPA5, 5′-AAGGUUACCCAUGCAGUUGTT-3′(siHSPA5–1), 5′-AGAUUCAGCAACUGGUUAAAGTT-3′ (siHSPA5–2), 5′-GAAAUCGAAAGGAUGGUUAAUTT-3′ (siHSPA5–3); ATF4 siRNA, 5-GCCUAGGUCUCUUAGAUGATT-3′ (siATF4–1), 5′-UCCCUCAGUGCAUAAAGGA-3′ (siATF4–2). ATF6 siRNA: 5′-UAAACUGAUAAUUGGUUGCdTdT-3′ (siATF6–1), 5′-UACACUUGUAGCUCACUCCCUGAGU-3′ (siATF6–2); PERK siRNA: 5′-UAAAGGUGCUUUCAAUAAAUCCGG-3′ (siPERK-1), 5′-GCAUCUGCCUGGUUACUUA-3′ (siPERK-2), 5′-CCAGAGAAGUGGCAAGAAA-dTdT-3′ (siPERK-3); IRE1α (ERN1) siRNA: 5′-GAUGUCCCACUUUGUGUCC-3′ (siIRE1–1), 5′-GGAGAGAAGCAGCAGACUU-3′ (siIRE1–2). Gene or siRNAs were transfected into cells using Lipofectamine 2000 reagent (11,668,019, ThermoFisher Scientific) as previously described [26].

### GPX4 activity assay

The activity of GPX4 was determined using a HT Glutathione Peroxidase Assay Kit (Trevigen, Inc. Gaithersburg, MD, USA). The assay principle was based on the oxidation of GSH to GSSG catalyzed by GPX4, which was then coupled to the recycling of GSSG back to GSH by glutathione reductase using NADPH as a reductant. The rate of decrease in NADPH absorbance measured at 340 nm during the oxidation of NADPH to NADP^+^ was directly proportional to GPX4 activity and thus was used to indicate GPX4 activity.

### Xenograft tumor model

All of the procedures of animal experiments were approved by the Ethics Committee of Zhengzhou University and performed in accordance with the Association for Assessment and Accreditation of Laboratory Animal Care guidelines (http://www.aaalac.org). Specific pathogen-free athymic nude BALB/c mice (4–6 weeks old) were obtained from Guangdong Experimental Animal Centre (Guangzhou, China). To generate murine subcutaneous tumors, cells (for U251: 2× 10^6^ cells; for U373: 2× 10^6^ cells) were suspended in 0.2 ml PBS and injected into the flanks of mice (*n* = 6/group). Tumor volume was measured once every 3 days using calipers. Tumor volume was estimated by the following formula: length × width^2^ × π/6 [28]. The mice whole body weight was measured every 2–3 days after inoculation. At the end of the experiment, all mice were killed by intraperitoneal injection of 200 mg/kg pentobarbital.

### Statistical analysis

Data were expressed as mean ± SD (standard deviation) from three independent experiments except animal experiments. Differences between groups were estimated using the Student’s *t*-test. All these analyses were performed using IBM SPSS Statistics 22.0 software (Armonk, NY, USA) and a two-tailed value of *P* < 0.05 was considered statistically significant.

For combination treatment of PERKi (PERK Inhibitor I, GSK2606414), EGCG and/or DHA, cell viability assay data were converted to fraction of growth affected by the individual drug or the combination treated cells compared with untreated cells and analyzed using CalcuSyn software (Biosoft, Ferguson, MO, USA) to determine whether the combination was synergistic. This program is based upon the Chou-Talalay equation [29], which calculates a combination index (CI). The general equation for the classic isobologram is given by: CI = (D)1/(Dx)1 + (D)2/(Dx)2. Where Dx indicates the dose of one compound alone required to produce an effect, (D)1 and (D)2 are the doses of compounds 1 and 2, respectively, necessary to produce the same effect in combination. From this analysis, the combined effects of the two compounds can be summarized as follows: CI < 1, CI = 1, CI > 1 indicate synergistic, additive and antagonistic effects, respectively [27].

## Results

### DHA showed anticancer effects in glioma cells

**Fig. 1:**
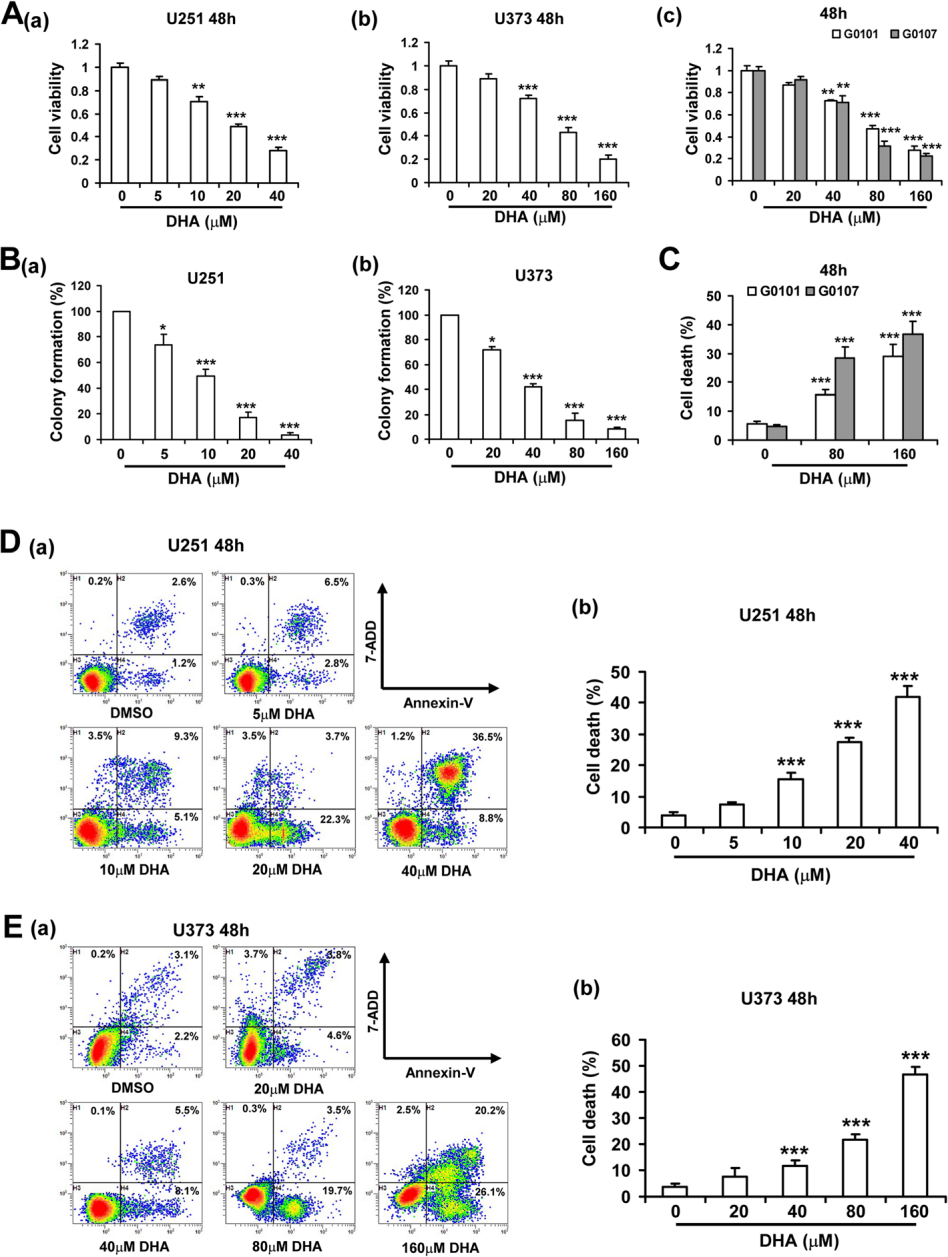
DHA reduced viability, colony formation and induced apoptosis in glioma cells. **A**, Cell viability of U251 (a), U373 (b) and primary human glioma cells (named G0101 and G0107) (c) was detected by using a Promega Cell Titer 96 Aqueous One Solution at 48 h after DHA treatment at different concentrations. **B**, DHA inhibited the colony formation of U251 (a) and U373 (b) cells. **C-E**, Cell death was detected by Annexin-V/7-ADD staining assay in human primary glioma cells (**C**), U251 (**D**) and U373 (**E**) cells treated with different concentrations DHA for 48 h, respectively. *, *P* < 0.05; **, *P* < 0.01; ***, *P* < 0.001, compared to control; Data were mean ± SD from three independent experiments. *n* = 3 for all bar graphs

We first assessed the effects of DHA on the proliferation of glioma cells. As shown in Fig. 1a, DHA treatment significantly inhibited the viability of both glioma cell lines (U251 and U373) and primary human glioma cells (G0101 and G0107) in a dose-dependent manner. Colony formation experiments further confirmed the anticancer effects of DHA in glioma cells (Fig. 1b). Moreover, flow cytometry analysis showed that DHA promoted cell death in both glioma cell lines and primary glioma cells (Fig. 1c-e). We further tested the effects of DHA on normal nerve cells and found that DHA had low toxicity on mouse HT22 nerve cells (Additional file 1: Figure S1).

### DHA induced ferroptosis in glioma cells

**Fig. 2:**
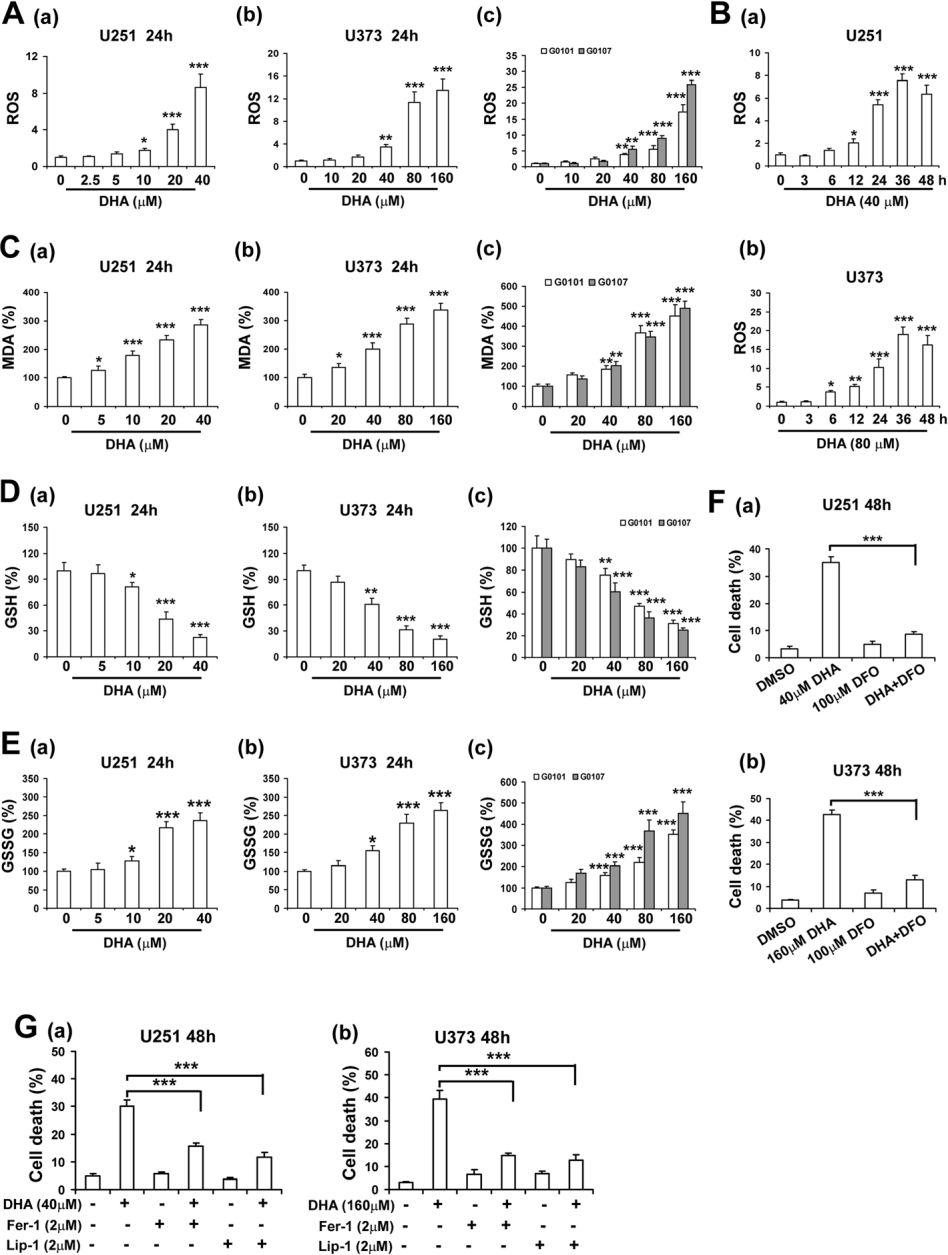
DHA induced ferroptosis in glioma cells. **A**, intracellular ROS levels in glioma cells treated with different concentrations of DHA. **B**, intracellular ROS levels in glioma cells at different time points after 40 μM DHA treatment. **C**, intracellular MDA levels in glioma cells treated with different concentrations of DHA. **D**, intracellular GSH levels in glioma cells treated with different concentrations of DHA. **E**, intracellular GSSG levels in glioma cells treated with different concentrations of DHA. **F**, iron chelator DFO inhibited DHA-induced glioma cell death. **G**, lipid peroxidation inhibitors reduced DHA-induced glioma cell death. *, *P* < 0.05; **, *P* < 0.01; ***, *P* < 0.001, compared to control. Data were mean ± SD from three independent experiments. *n* = 3 for all bar graphs

Although previous studies have shown that iron-mediated ROS generation is involved in the cytotoxicity of DHA towards cancer cells [30], the capacity of DHA in ferroptosis induction is underestimated. We therefore determined whether DHA induced ferroptosis in glioma cells. As shown in Fig. 2a and b, DHA significantly increased intracellular ROS production in U251 and U373 cells as well as primary glioma cells in a dose- and time-dependent manner. Given lipid peroxidation is the key event during ferroptosis, we further investigated the effects of DHA on lipid ROS and MDA, an end product of lipid peroxidation in glioma cells. As shown in Additional file 1: Figure S2 and S2C, DHA significantly increased lipid ROS and MDA levels in glioma cells in a dose- and time-dependent manner. As an important antioxidant, reduced form GSH was exhausted by DHA (Fig. 2d), while oxidized form GSSG was accumulated in glioma cells (Fig. 2e). Since ferroptosis is an iron-dependent cellular death, we assessed whether iron chelator deferoxamine (DFO) affected the anticancer effects of DHA. As shown in Additional file 1: Figure S3A, S4 and 2F, DFO significantly abolished DHA-induced ROS, lipid ROS and MDA generation as well as ferroptosis in glioma cells. Moreover, lipid peroxidation inhibitors ferrostatin-1 (Fer-1) and liproxstatin-1 (Lip-1) also inhibited DHA-induced ROS, lipid ROS and MDA generation as well as ferroptosis in both glioma cell lines (Additional file 1: Figure S3B, S4 and S2G), verifying the pro-ferroptotic effects of DHA.

### Unfolded protein response attenuated anti-tumor effects of DHA via PERK/ATF4 pathway

**Fig. 3:**
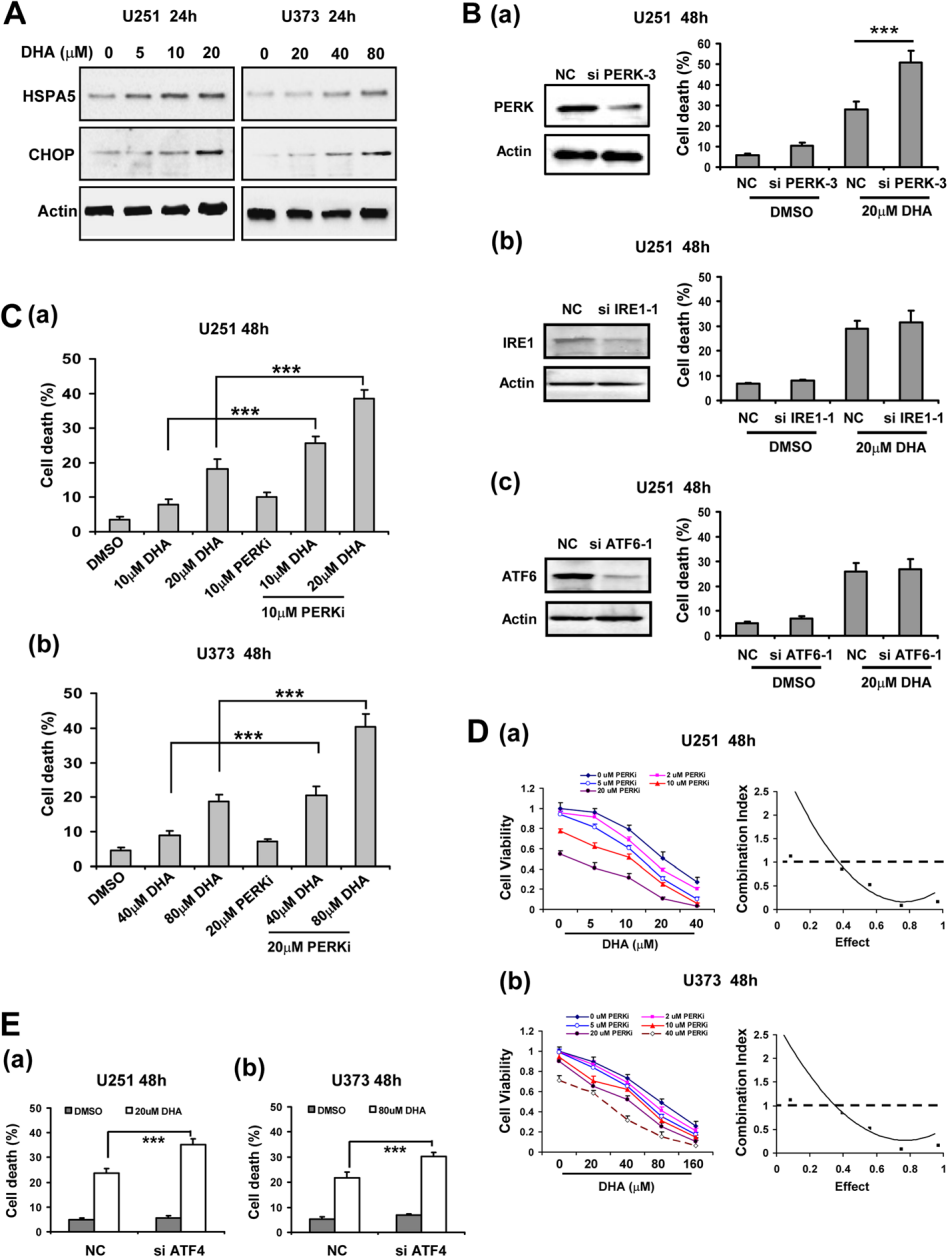
Unfolded protein response attenuated anti-glioma effects of DHA via PERK/ATF4 pathway. **A**, expression of ER stress biomarker HSPA5 and CHOP in glioma cells treated with DHA at different concentrations. **B**, DHA-induced glioma cell death after silencing PERK, IRE1 or ATF6. **C**, ER stress inhibitor PERKI enhanced DHA-induced glioma cell death. **D**, PERKI synergistically enhanced anti-gliomaeffects of DHA. **E**, ATF4 silencing enhanced DHA-induced glioma cell death. ***, *P* < 0.001, compared to control. Data were mean ± SD from three independent experiments. *n* = 3 for all graphs

Intracellular and extracellular insults can cause the accumulation of misfolded proteins at the ER lumen, a condition termed ER stress that would trigger unfolded protein response (UPR) and endows cancer cells with greater tumorigenic and drug-resistant capacity [11, 23]. ER stress can be triggered by anticancer chemicals, including pro-ferroptotic reagents such as erastin [17]. Like erastin, DHA also induced ER stress in a dose-dependent manner in glioma cells, as indicated by gradually elevated expression of HSPA5 and CHOP proteins (Fig. 3a). During ER stress, UPR signaling can be mediated by three ER-transmembrane sensors, PERK, IRE1α and ATF6 [23]. We thus tested whether these signal pathways affect DHA-induced ferroptosis in glioma cells. As shown in Fig. 3b and Additional file 1: Figure S5, silencing PERK, but not IRE1α or ATF6α significantly enhanced DHA-induced cell death in glioma cells. Moreover, PERKi (PERK Inhibitor I, GSK2606414) also significantly enhanced DHA-induced cell death in glioma cells, suggesting that PERK pathway may attenuate the cytotoxicity of DHA. To evaluate the potential synergistic anticancer effects of DHA and PERKi, glioma cells were treated with increasing doses of DHA and PERKi alone or in combination, and effects on cell death were assessed. As shown in Fig. 3c, combination of DHA and PERKi significantly promoted glioma cell death than either drug alone. These data implied that the DHA and PERKi might synergize to enhance cell death in glioma cells. To confirm this synergism, glioma cells were treated with a combination of the two compounds in a constant ratio to one another, cell viability was detected and combination index (CI) was calculated. As shown in Fig. 3d, significant synergies between the two agents (CI < 1) were found in both glioma cell lines. ATF4 (activating transcription factor 4) is central to PERK-governed signaling and its translation is activated upon the phosphorylation of eIF2α (eukaryotic translation initiation factor 2α) by PERK. As shown in Fig. 3e, silencing of ATF4 by siRNA significantly enhanced DHA-induced cell death like PERKi in U251 and U373 cells. These data suggested that PERK/ATF4 pathway might attenuate DHA-induced ferroptosis in glioma cells.

### Inhibition of PERK/ATF4 signaling enhanced DHA-induced ferroptosis in glioma cells

**Fig. 4:**
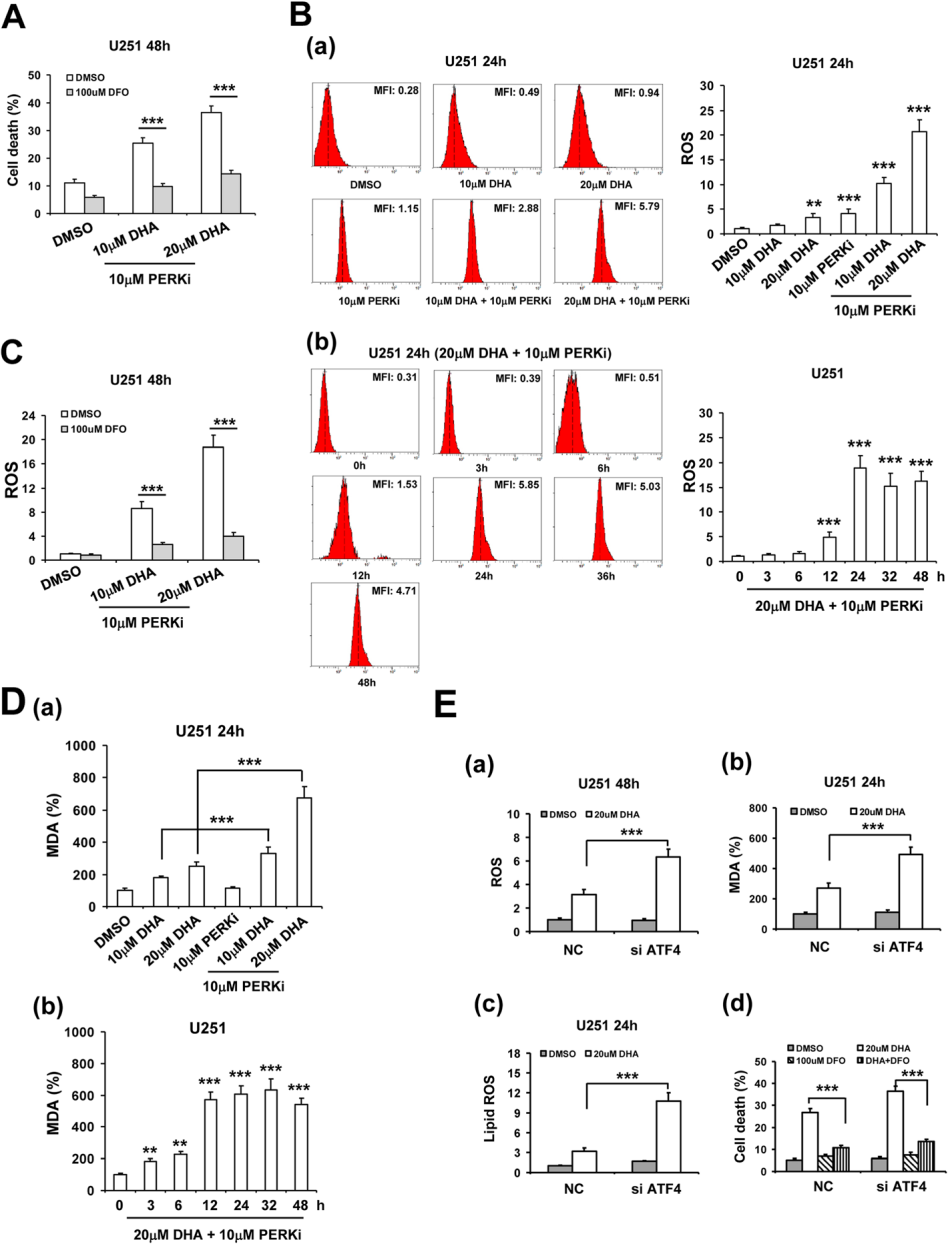
Inhibition of PERK-ATF4 signaling enhanced DHA-induced ferroptosis in glioma cells. **A**, DFO inhibited the synergistic effects on glioma cell death of DHA combined with PERKI. **B**, (a) PERKi enhanced DHA-induced U251 cell ROS generation. (b) time course of ROS generation in U251 cells treated with 20 μM DHA combined with 10 μM PERKi. **C**, DFO suppressed the synergistic effects on ROS production of DHA combined with PERKi. **D**, (a) PERKi enhanced DHA-induced U251 cell MDA generation. (b) Time course of MDA generation in U251 cells treated with 20 μM DHA combined with 10 μM PERKi. **E**, ATF4 siRNA enhanced DHA-induced ROS (a), MDA (b) and lipid ROS (c) generation in glioma cells as well as cell death (d). *, *P* < 0.05; **, *P* < 0.01; ***, *P* < 0.001, compared to control. Data were mean ± SD from three independent experiments. *n* = 3 for all bar graphs

Since PERK/ATF4 signaling mediated DHA-induced UPR in glioma cells, we investigated whether PERK/ATF4 inhibition enhanced anticancer activity of DHA through ferroptosis induction. Our results showed that PERKi significantly enhanced DHA-induced cell death in glioma cells (Figs. 4a and Additional file 1: Figure S6A), which could be inhibited by DFO. Moreover, DFO could also abolish PERKi-enhanced ROS generation in DHA-treated U251 (Fig. 4b-c) and U373 cells (Additional file 1: Figure S6B-C). Simultaneously, PERKi also enhanced DHA-induced MDA production in glioma cells (Figs. 4d and Additional file 1: Figure S6D), suggesting the important role of ferroptosis in the synergistic anticancer effects of PERKi and DHA combination. In addition, silencing of ATF4 by siRNA showed similar synergistic pro-ferroptotic effects with DHA in glioma cells, as indicated by enhanced ROS, MDA and lipid ROS production as well as cell death (Fig. 4e and Additional file 1: Figure S6E). In contrast, ATF4 overexpression significantly attenuated DHA-induced ROS, MDA and lipid ROS production as well as cell death (Additional file 1: Figure S7).

### ATF4-induced HSPA5 expression protected glioma cells against DHA-induced ferroptosis

**Fig. 5:**
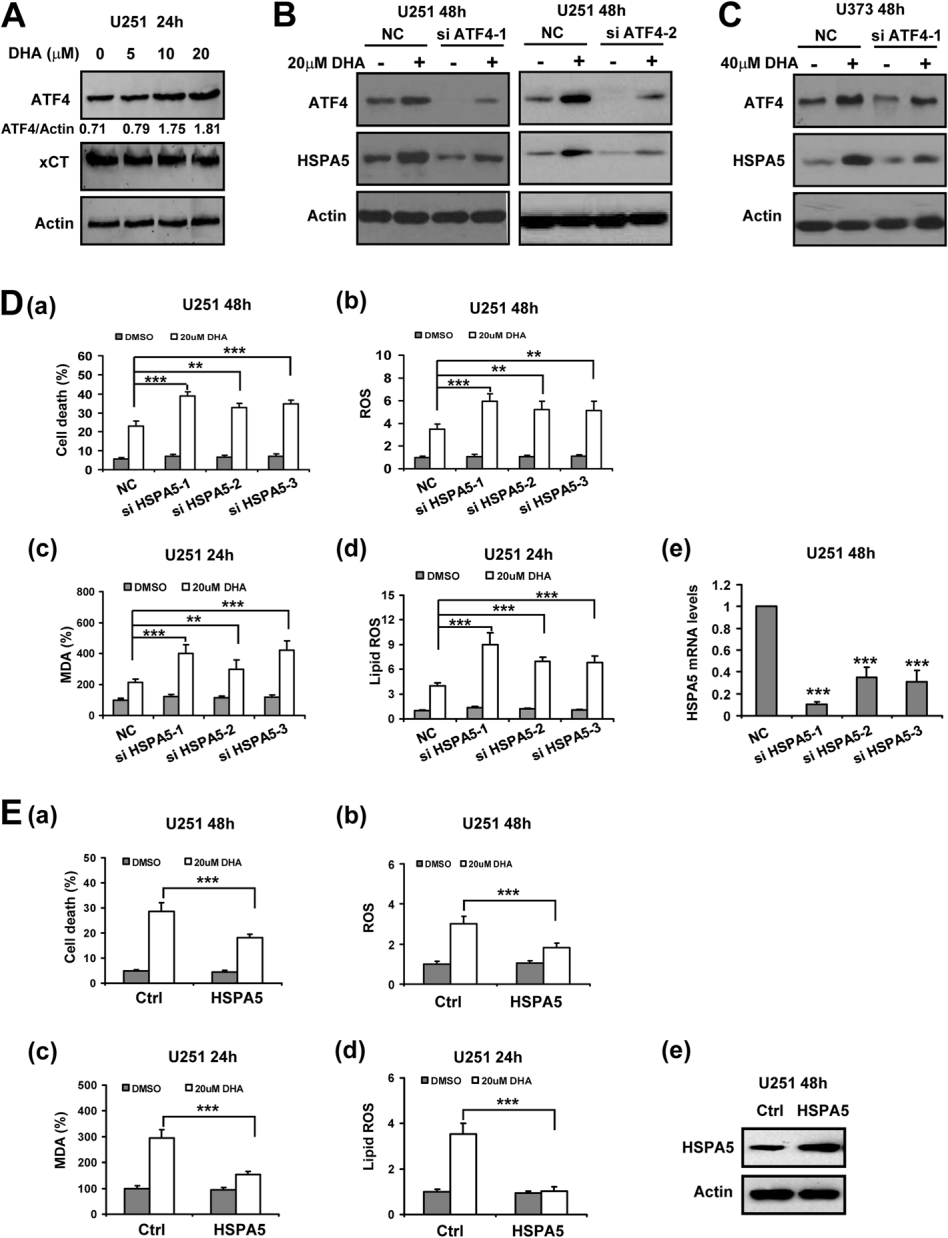
ATF4-induced HSPA5 prevented DHA-induced ferroptosis in glioma cells. **A**, ATF4 and xCT expression in U251 cells 24 h after DHA treatment at different concentration. The relative expression level of ATF4 standardized to β-actin was labeled. **B** and **C**, ATF4 silencing inhibited DHA-induced HSPA5 expression in glioma cells. **D**, HSPA5 silencing (e) enhanced DHA-induced glioma cell death (a), and ROS (b), MDA (c) and lipid ROS (d) generation. **E**, HSPA5 overexpression (e) inhibited DHA-induced glioma cell death (a), and ROS (b), MDA (c) and lipid ROS (d) generation. **, *P* < 0.01; ***, *P* < 0.001, compared to control. Data were mean ± SD from three independent experiments. *n* = 3 for all bar graphs

During ER stress, ATF4 is selectively translated to promote the expression of molecules that favor the adaptive survival of cancer cells such as SLC7A11/xCT [31]. However, xCT expression was not increased in DHA-treated glioma cells whereas enhanced ATF4 expression was induced (Fig. 5a). Since HSPA5 is a master chaperone that could be upregulated by ATF4 during ER stress [32], we examined the impact of ATF4 knockdown on HSPA5 expression in DHA-treated glioma cells. Notably, silencing of ATF4 expression using siRNA inhibited DHA-induced HSPA5 expression in both U251 and U373 cells (Fig. 5b and c). Moreover, suppression of HSPA5 expression by siRNA significantly increased ROS, MDA and lipid ROS levels as well as cell death in glioma cells treated with DHA (Fig. 5d and Additional file 1: Figure S8A). In contrast, overexpression of HSPA5 inhibited DHA-induced ROS, MDA and lipid ROS generation and cell death in glioma cells (Fig. 5e and Additional file 1: Figure S8B), suggesting that ATF4-induced HSPA5 might antagonize the pro-ferroptotic activity of DHA.

### Inhibitor of HSPA5 synergistically enhanced anti-tumor effects of DHA

**Fig. 6:**
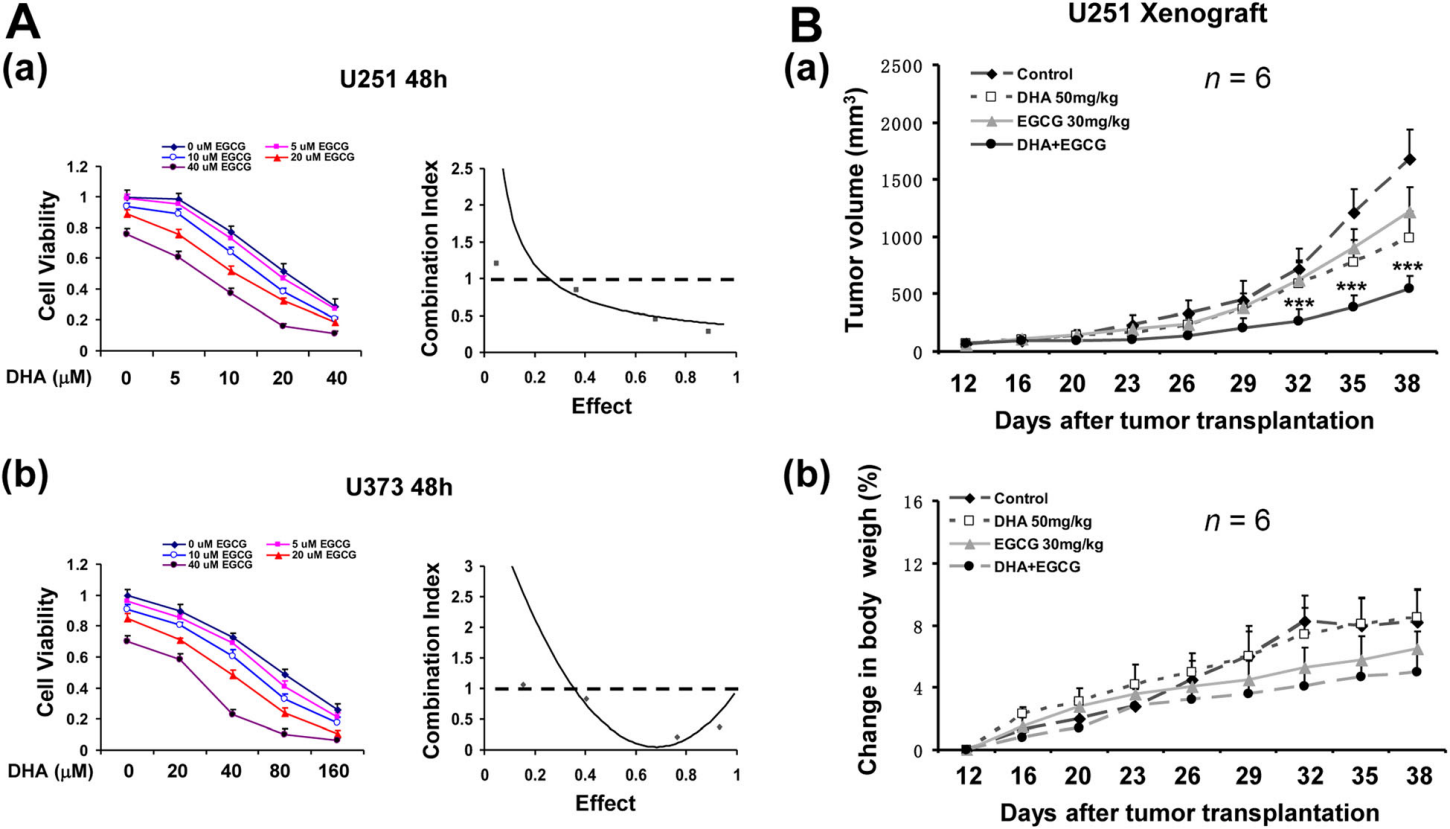
HSPA5 inhibitor synergistically enhanced the anti-glioma effects of DHA. **A**, HSPA5 inhibitor EGCG synergistically enhanced anti-tumor effects of DHA cells in vitro. Data were mean ± SD from three independent experiments (*n* = 3). **B**, EGCG synergistically enhanced anti-tumor effects of DHA cells in mouse xenograft glioma model. ***, *P* < 0.001, compared to control

Since HSPA5 protected glioma cells from DHA-induced ferroptosis, we hypothesized that HSPA5 inhibition could enhanced the anticancer capacity of DHA. As shown in Fig. 6a, HSPA5 inhibitor EGCG showed similar synergistic anticancer effects with DHA. To further test the synergistic effects of DHA and ER stress inhibition in vivo, we evaluated the antitumor activity of DHA/EGCG in glioma xenografts in nude mice. As shown in Fig. 6b, while either DHA or EGCG alone inhibited the tumors growth, their combination exerted a much stronger antitumor effects in U251 xenograft tumor models (*P* < 0.01). In addition, no significant increase in body weight loss was observed in the mice treated with the drugs, suggesting that the side effects of the two drugs were minimal in vivo.

### HSPA5 antagonized DHA-induced ferroptosis by increasing GPX4 in glioma cells

**Fig. 7:**
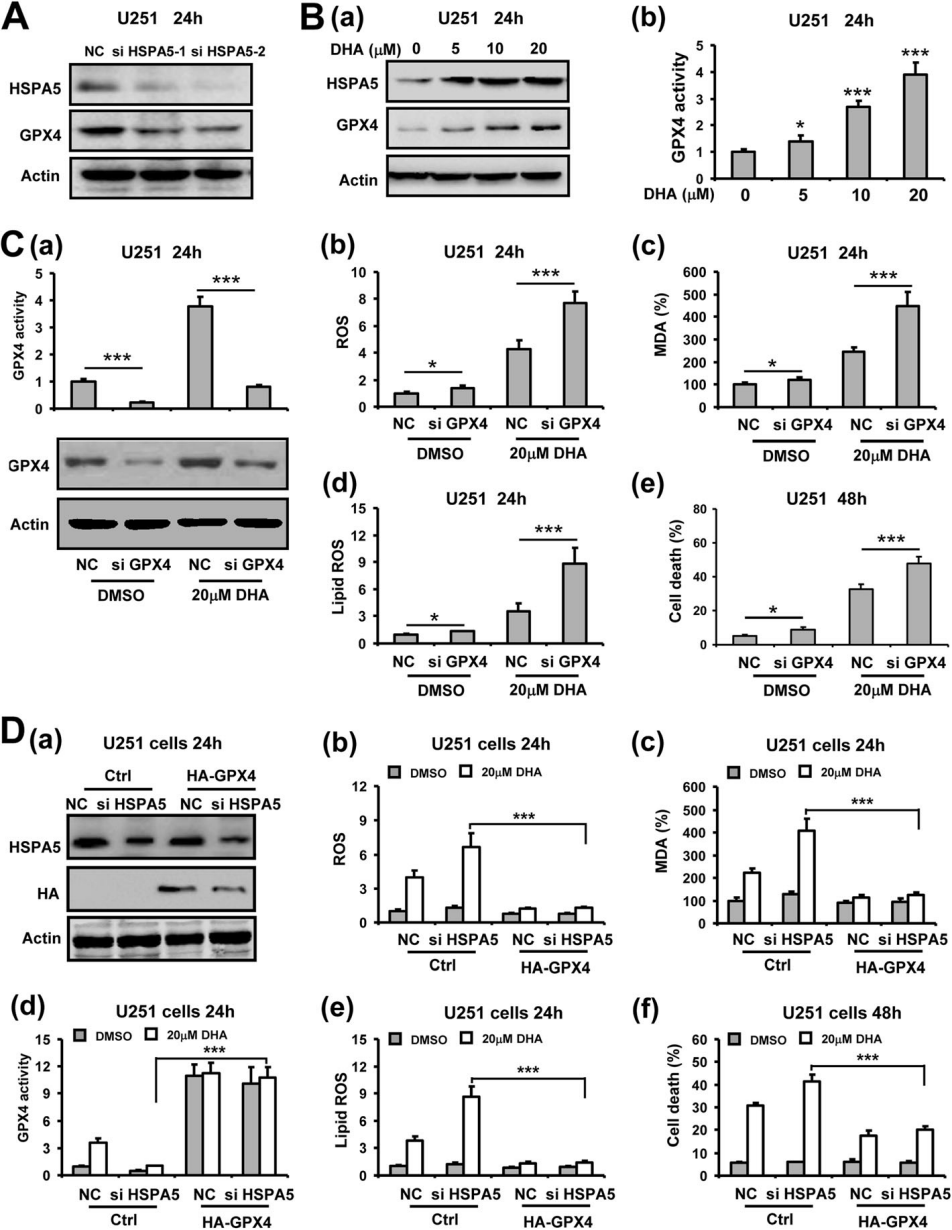
HSPA5 inhibited DHA-induced ferroptosis by increasing GPX4 in glioma cells. **A**, GPX4 expression after HSPA5 silencing in U251 cells. **B**, GPX4 expression (a) and activity (b) in U251 cells treated with DHA at different concentrations. **C**, Inhibition of GPX4 activity by siRNA transfection (a) enhanced DHA-induced ROS (b), MDA (c) and lipid ROS (d) generation as well as cell death (e) in U251 cells. **D**, GPX4 overexpression (a) compensated HSPA5 siRNA-enhanced reduction of GPX4 activity (d) and abolished HSPA5 siRNA-enhanced ROS (b), MDA (c) and lipid ROS (e) generation and cell death (f) in U251 cells. *, *P* < 0.05; **, *P* < 0.01; ***, *P* < 0.001, compared to control. Data were mean ± SD from three independent experiments. *n* = 3 for all bar graphs

Given that GPX4 is the critical enzyme that reduces lipid peroxidation to prevent ferroptosis [16], we thus assessed the roles of GPX4 in HSPA5-mediated ferroptotic resistance against DHA in glioma cells. As shown in Fig. 7a, silencing HSPA5 by siRNAs suppressed GPX4 expression in glioma cells. Synchronous with HSPA5 upregulation, our Western blot analysis showed that both expression and activity of GPX4 were elevated by DHA in a dose-dependent manner (Fig. 7b), indicating that HSPA5 may activate GPX4 under ER stress of ferroptosis. Moreover, downregulating GPX4 using siRNA remarkably enhanced DHA-induced ROS, MDA and lipid ROS production as well as cell death rate in U251 cells (Fig. 7c). We next restored GPX4 activity in glioma cells using an HA-tagged GPX4 expression vector in HSPA5-silenced glioma cells. Notably, GPX4 restoration significantly counteracted with the enhancement of DHA-induced ROS, MDA and lipid ROS production as well as cell death rate by HSPA5 silencing in both U251 and U373 cells (Fig. 7d and Additional file 1: Figure S9), suggesting that GPX4 mediated the anti-ferroptotic effects of HSPA5 against DHA in glioma cells.

## Discussion

**Fig. 8:**
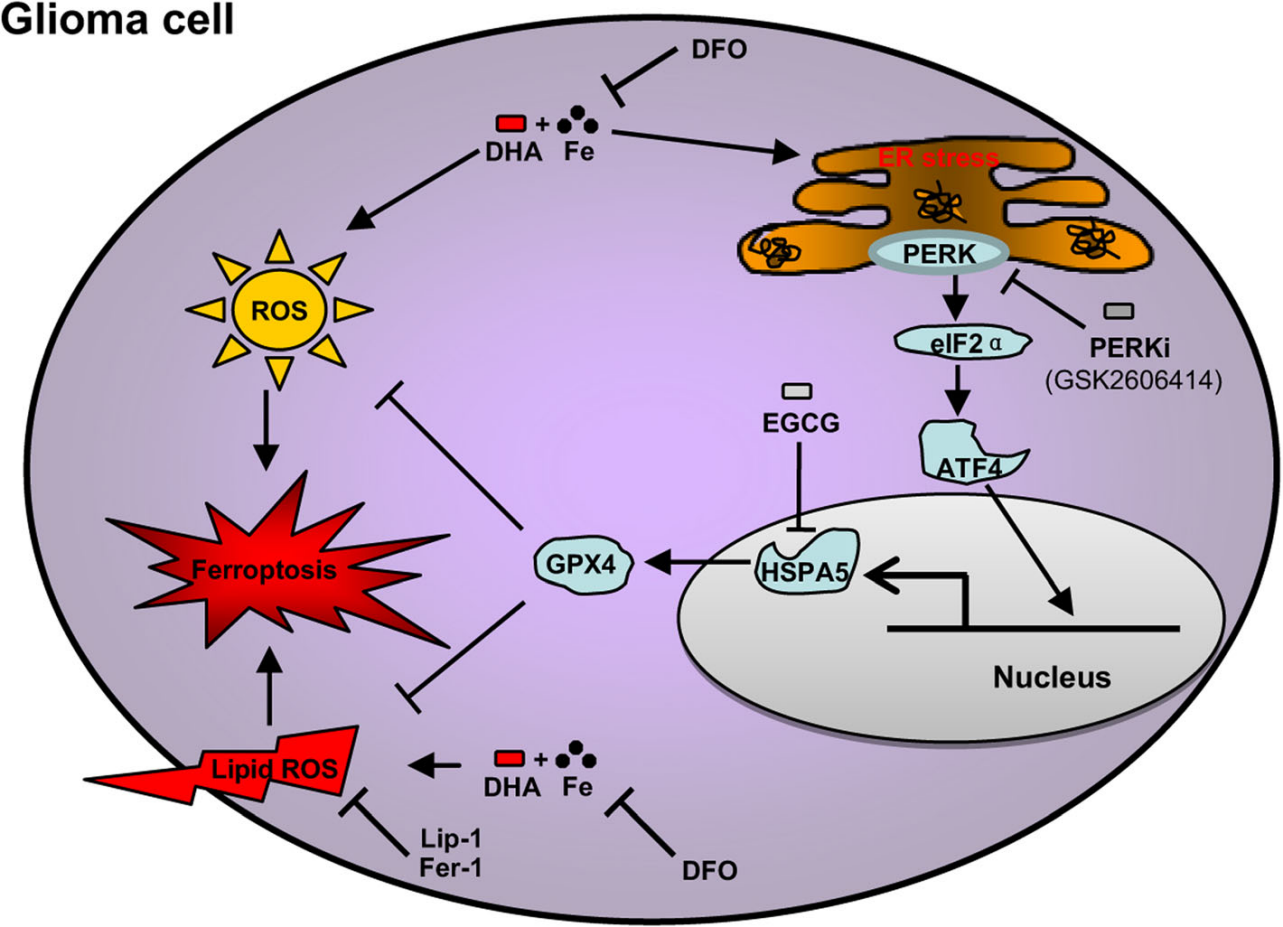
Schematic depicting HSPA5 protects against DHA-induced ferroptosis in glioma cells

In this study, we investigated the anticancer effects of DHA in glioma cells. We for the first time demonstrated that DHA induced glioma cell death through triggering ferroptosis in both dose- and time-dependent manners. Moreover, we found that DHA induced-ER stress in turn activated cell protection against ferroptosis through PERK-ATF4- HSPA5 activation, which promoted the expression of GPX4 to detoxify peroxidized membrane lipids (Fig. 8). Furthermore, inhibition of PERK, ATF4 or HSPA5 enhanced cytotoxic effects of DHA on glioma cells both in vivo and in vitro. These findings revealed a novel anticancer aspect of DHA and its negative feedback regulatory pathway in glioma cells.

Besides to its antimalarial usage, DHA has been considered as a promising anticancer reagent for decades. The anticancer activity of DHA has been shown to involve a series of cellular procedures, including oxidative stress response, DNA damage and repair, cell death induction, inhibition of metastasis and angiogenesis, as well as pro-oncogenic signaling suppression [30]. Although the mechanisms by which DHA induces cell death have not been fully elucidated, iron-dependent ROS generation by cleavage of endoperoxide moiety has been accepted as the crucial procedure for its cytotoxicity [30]. Moreover, treatment with either ferrous iron or transferrin can enhance the cytotoxicity of DHA toward cancer cells, which could be reversed by iron chelator or anti-transferrin receptor antibody [33, 34]. These findings strongly hint that DHA may exert an anticancer effect through ferroptosis. Indeed, several studies have shown that ART derivates including DHA can induce ferroptosis in head and neck carcinoma as well as ovarian cancer cells [15, 35]. In line with these finding, we for the first time demonstrated that DHA induced ferroptosis in glioma cells, based on the increased ROS generation and lipid peroxidation as well as GSH exhaustion, which could be inhibited by the iron complexant DFO or lipid peroxidation inhibitor ferrostatin-1 or liprostatin-1. These data suggest that ferroptosis may be an important anticancer mechanism of DHA. In contrast, most studies up-to-date have shown that DHA induce apoptosis in cancer cells. For example, Qin and colleagues have found that DHA induces loss of mitochondrial membrane potential, cytochrome c release, caspase activation and phosphatidylserine externalization in liver cancer cells [36]. Other studies have demonstrated that DHA can regulate various signaling pathway to mediate caspase-dependent and -independent apoptosis [37–39]. Therefore, whether DHA-induced ferroptosis and apoptosis share some common regulatory mechanisms and when they separate each other need further investigation.

ER stress is a condition in which misfolded/unfolded proteins accumulate in the ER lumen, which overwhelms cellular proteostasis. Numerous factors can cause ER stress, including oncogene activation, hypoxia, nutrient deprivation, acidosis, anticancer treatment and oxidative imbalance [23]. As a strong oxidative source, ferroptosis has been shown to induce ER stress in several human cancer cells [17, 18, 39]. As a result of accumulation of misfolded/unfolded proteins, UPR is activated through PERK, IRE1α and ATF6 pathways to restore proteostasis and oxidative balance. In this study, we found that inhibition of PERK by either siRNA or PERKi significantly enhanced DHA-induced lipid peroxidation and glioma cell death, which could be abolished by DFO. However, IRE1α or ATF6 silencing did not enhance DHA-induced glioma cell death. These findings suggest that UPR through PERK pathway may protect glioma cells from DHA-induced ferroptosis. However, the biological roles of IRE1α and ATF6 signaling in DHA-induced ER stress need further investigation.

As a key transcription factor of ER stress signaling, ATF4 translation is selectively enhanced by phosphorylated eIF2α after PERK activation [40]. ATF4 controls the expression of genes that promote cell survival and stress adaption, including amino acid transport and metabolism, antioxidant response, autophagy and protein folding [41]. Tumor cells frequently exploit ATF4 to reduce the stress resulting from rapid proliferation and nutrient limitation inside a growing tumor mass [42]. Previous studies have demonstrated that ATF4 is involved in chemoresistance via transcriptional regulation of membrane transporters and enzymes required for GSH biosynthesis in cancer cells [31]. Moreover, PERK-mediated ATF4 activation has exhibited protective roles against erastin-induced ferroptosis in pancreatic cancer cells [39]. In line with these findings, we found that ATF4 silencing significantly enhanced DHA-induced lipid peroxidation and glioma cell death like PERKi, suggesting that PERK-induced ATF4 activation may exert a protective effect against ferroptosis in glioma cells. Although most of its target genes are involved in various salvage pathways that promote cell survival, ATF4 can also exert tumor-suppressive effects through inducing apoptosis, cell cycle arrest and senescence under persistent unresolved stress conditions [43], suggesting ATF4 might play dual role in cell death regulation possibly in a context-dependent manner. Further investigations are needed to elucidate how ATF4 switches its functions between cancer protection against cell death and tumor suppression in specific context.

Under ER stress, a wide range of enzymes and chaperons are induced to exert cellular protection. HSPA5, as a prominent chaperone protein that is overexpressed in a wide range of cancer cells [24], can be induced under ER stress conditions such as lipid peroxidation and promote cancer progression and therapy resistance [44]. HSPA5 transcription can be activated by the binding of its ATF/CRE sequence with ATF4 [32]. In this study, we found DHA-induced HSPA5 expression could be attenuated by ATF4 silencing, indicating that ATF4 may promote HSPA5 expression under DHA-induced ER stress. Moreover, HSPA5 silencing enhanced DHA-induced ferroptosis, while HSPA5 overexpression had an opposite effect in glioma cells, which is similar to the fidings in pancreatic cancer cells [17]. These data suggested that ATF-induced HSPA5 upregulation might be a crucial molecule for cell adaption in ER stress caused by ferroptosis.

Accumulation of lipid peroxidation products is recognized as a central mediator of ferroptosis [6]. GPX4, the only peroxidase that eliminates lipid peroxides and protects membrane fluidity using GSH as its cofactor, has been implicated as an essential regulator of ferroptosis [45]. Previous studies have demonstrated that direct inhibition of GPX4 using RSL3 or GPX4 knockdown can cause ferroptosis [16]. Moreover, some ferroptosis inducers such as erastin and FIN56 have been shown to cause GPX4 degradation [46, 47]. In contrast, we found that DHA increased GPX4 expression by HSPA5 upregulation to exert a cell-protective effect against ferroptosis, while silencing GPX4 by siRNA enhanced the pro-ferroptotic effect of DHA. These data are similar to the findings of in erastin-treated pancreatic cancer cells [17], suggesting that ER stress-induced GPX4 activation by HSPA5 might serve as a negative feedback loop during ferroptosis.

Despite great advances in combined treatment regimen consisting of maximum resection with concurrent chemoradiotherapy, malignant glioma remains a highly lethal disease [2]. Temozolomide, an alkylating agent, is widely used as the first-line chemotherapeutic drug for glioblastoma treatment [42]. However, the efficacy of temozolomide is often limited by the development of resistance [48]. Recent studies have demonstrated that ferroptosis inducers foster the cytotoxicity of temozolomide toward glioma cells [49], implying that ferroptosis inducers such as DHA could enhanced the antitumor effects of temozolomide. Previous studies have demonstrated that HSPA5 expression is elevated in glioma specimens and silencing HSPA5 increases temozolomide chemosensitivity in malignant glioma cell lines [50], suggesting the potential roles of UPR in glioma chemoresistance. Considering the crucial roles of PERK, ATF4 and HSPA5 during UPR and subsequent anti-ferroptotic effects, it is reasonable to enhance anticancer effects by targeting UPR pathways. In this study, we found that either inhibiting or silencing PERK, ATF4 or HSPA5 could enhance the pro-ferroptotic effects of DHA. However, in the absence of DHA, knockdown of these three genes did not observably induce cell death and the production of ROS and MDA in glioma cells. This could be explained by the fact that the anticancer effects of DHA relies on carbon-centered free radicals and ROS generated by the reaction of its endoperoxide bond with iron [34]. In the absence of DHA, the basal levels of ROS and MDA in glioma cells are very low and could not be potentiated by knockdown of PERK, ATF4 and HSPA5. Moreover, UPR is activated and exerts cell protection under ER stress such as DHA treatment instead of normal conditions, which is in line with previous findings [17, 51, 52]. Therefore, interfering this negative feedback by siRNA or small molecules such as PERKi or EGCG would be a promising strategy to enhance the anticancer efficacy of ferroptosis inducers, including DHA.

## Conclusions

In summary, we provided the evidence of ferroptosis as a novel anticancer mechanism of DHA in glioma, which could be neutralized by PERK-ATF4-HSPA5-GPX4 pathway activation under ER stress. Therefore, inhibition ER stress or subsequent PERK-ATF4-HSPA5-GPX4 pathway might enhance the anticancer activity of DHA by increasing ferroptosis in glioma.

## Supplementary information

**Additional file 1: Figure S1.** DHA had low toxicity in mouse HT22 normal nerve cells. Cell death (A) and viability (B) were not affected by DHA at different concentrations. **Figure S2.** DHA induced ferroptosis in glioma cells. **Figure S3.** Ferroptosis inhibitor reduced DHA-induced cell death in primary glioma cells. **Figure S4.** Iron chelator DFO and lipid peroxidation inhibitors reduced DHA-induced ROS (A), MDA (B) and lipid ROS (C) generation. **Figure S5.** DHA-induced glioma cell death after silencing PERK (A & B), IRE1 (C) and ATF6 (D). **Figure S6.** Inhibition of PERK/ATF4 signal enhanced DHA-induced ferroptosis of U373 cells. **Figure S7.** Effects of ATF4 overexpression (E) on DHA-induced ROS, lipid ROS, and MDA generation as well as cell death in glioma cells. **Figure S8.** ATF4-induced HSPA5 prevented DHA-induced ferroptosis in U373 cells. **Figure S9.** HSPA5 protected against DHA-induced ferroptosis by increasing GPX4 in U373 cells.

## Data Availability

Please contact the corresponding author for all data requests.

## Abbreviations

ART: Artemisinin
CHOP: CCAAT/enhancer-binding protein homologous protein
CI: Combination index
DFO: Deferoxamine
DHA: Dihydroartemisinin
EGCG: Epigallocatechine gallate
ER: Endoplasmic reticulum
GPX4: Glutathione peroxidase 4
HSPA5: Heat shock protein family A (Hsp70) member 5
IRE1α: Inositol-requiring transmembrane kinase/endoribonuclease 1α
MDA: Malondialdehyde
NADPH: Reduced β-nicotinamide adenine dinucleotide phosphate
NC: Negative control
PERK: Protein kinase R-like ER kinase
ROS: Reactive oxygen species
UPR: Unfolded protein response
