# Supplementary material for: Dihydroartemisinin-induced unfolded protein response feedback attenuates ferroptosis via PERK/ATF4/HSPA5 pathway in glioma cells
Source: J Exp Clin Cancer Res. 2019 Sep 13;38:402. doi: 10.1186/s13046-019-1413-7 (PMC6743121; doi:10.1186/s13046-019-1413-7)
Supplement: Supplementary file 1 — Additional file 1: Figure S1. DHA had low toxicity in mouse HT22 normal nerve cells. Cell death (A) and viability (B) were not affected by DHA at different concentrations. Figure S2. DHA induced ferroptosis in glioma cells. Figure S3. Ferroptosis inhibitor reduced DHA-induced cell death in primary glioma cells. Figure S4. Iron chelator DFO and lipid peroxidation inhibitors reduced DHA-induced ROS (A), MDA (B) and lipid ROS (C) generation. Figure S5. DHA-induced glioma cell death after silencing PERK (A & B), IRE1 (C) and ATF6 (D). Figure S6. Inhibition of PERK/ATF4 signal enhanced DHA-induced ferroptosis of U373 cells. Figure S7. Effects of ATF4 overexpression (E) on DHA-induced ROS, lipid ROS, and MDA generation as well as cell death in glioma cells. Figure S8. ATF4-induced HSPA5 prevented DHA-induced ferroptosis in U373 cells. Figure S9. HSPA5 protected against DHA-induced ferroptosis by increasing GPX4 in U373 cells. [file 13046_2019_1413_MOESM1_ESM.doc]

**Additional file**


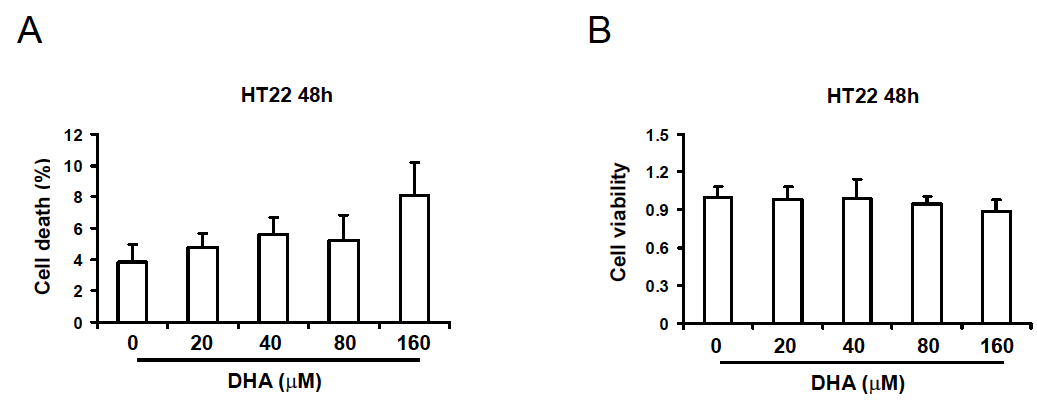


**Figure S1.** DHA had low toxicity in mouse HT22 normal nerve cells. Cell death (A) and viability (B) were not affected by DHA at different concentrations. Data were mean ± SD from three independent experiments. *n* = 3 for all bar graphs.


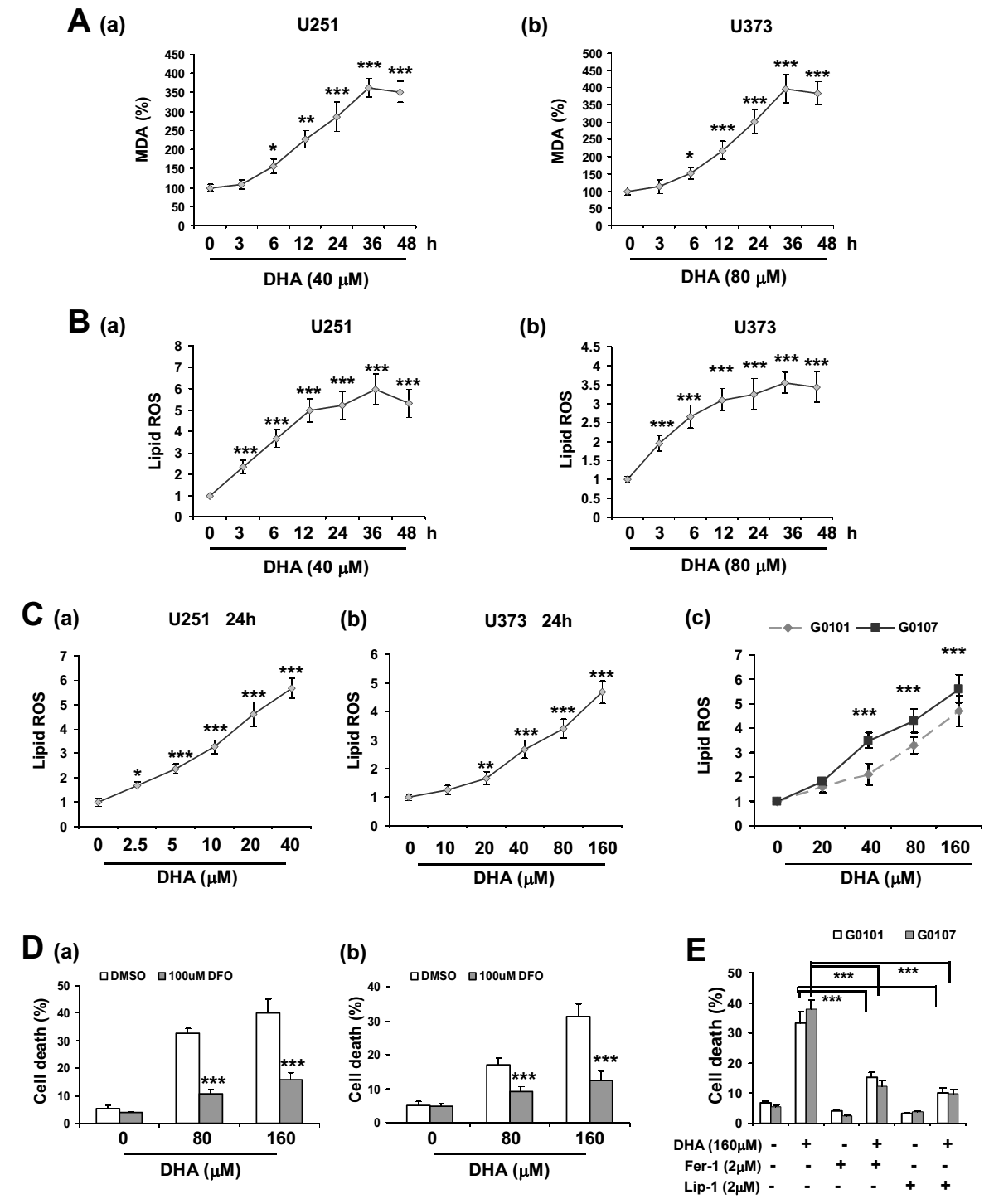


**Figure S2.** DHA induced ferroptosis in glioma cells. A, intracellular MDA levels in U251 (b) and U373 (b) at different time points after 40 μM and 80 μM DHA treatment, respectively. B, lipid ROS levels in U251 (b) and U373 (b) at different time points after 40 μM and 80 μM DHA treatment, respectively. C, lipid ROS levels in glioma cells treated with different concentrations of DHA.


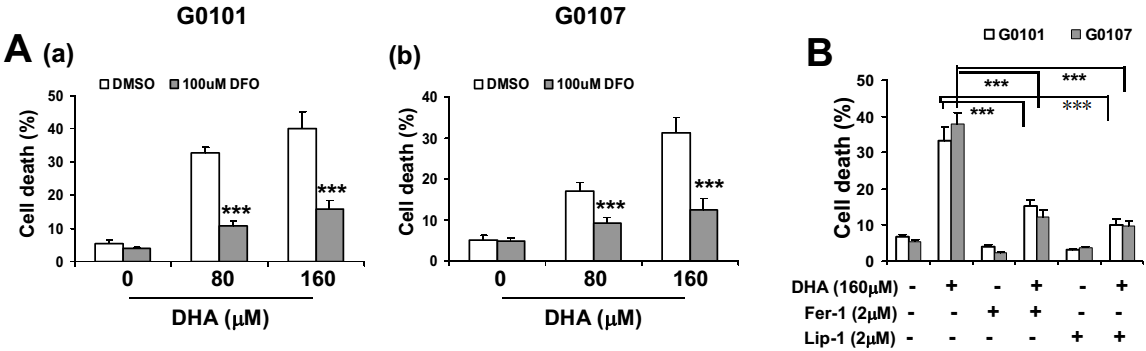


**Figure S3.** Ferroptosis inhibitor reduced DHA-induced cell death in primary glioma cells. A, iron chelator DFO inhibited DHA-induced cell death in glioma cells. B, lipid peroxidation inhibitors reduced DHA-induced glioma cell death ***, *P* < 0.001, compared to control. Data were mean ± SD from three independent experiments. *n* = 3 for all graphs.


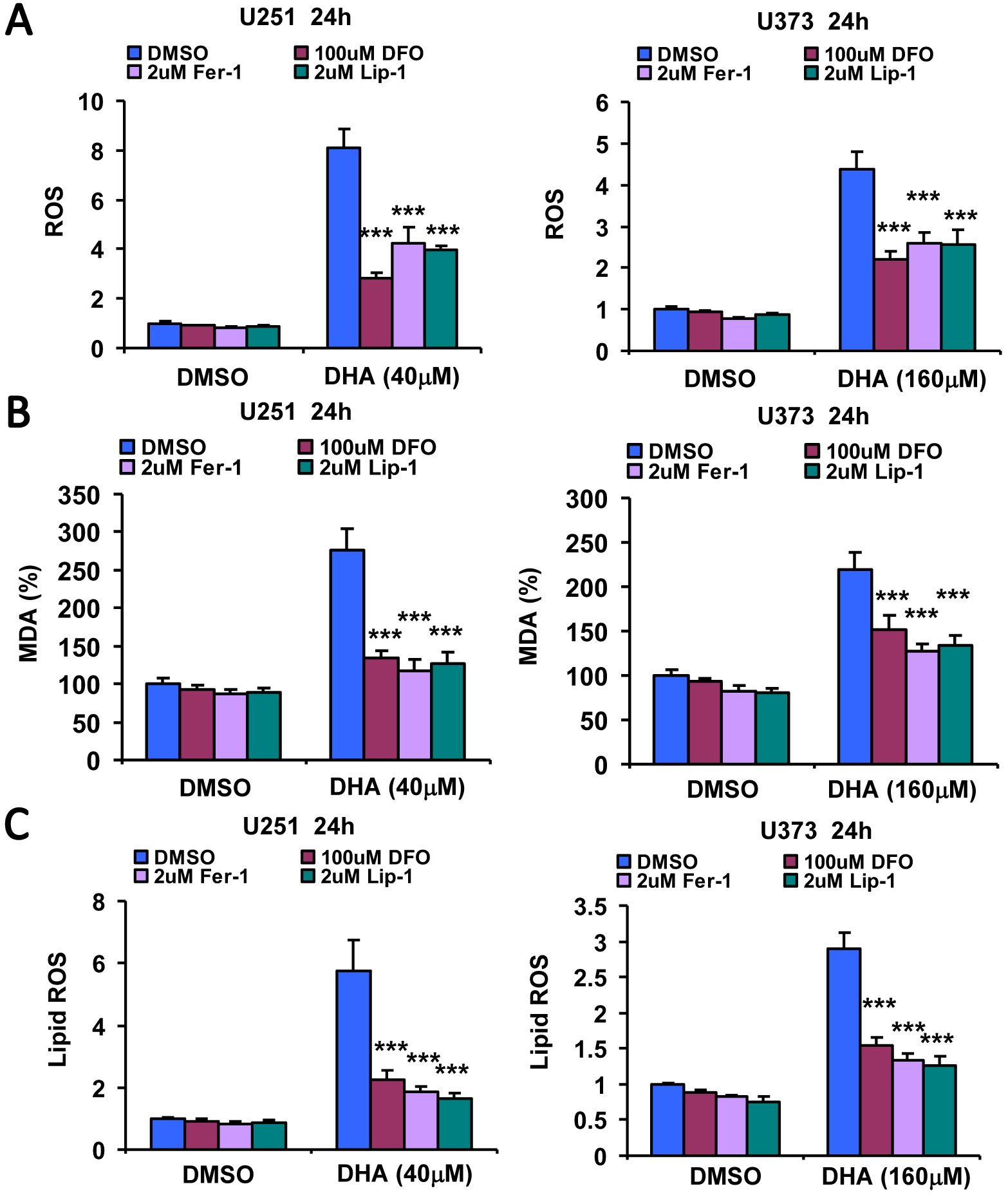


**Figure S4.** Iron chelator DFO and lipid peroxidation inhibitors reduced DHA-induced ROS (A), MDA (B) and lipid ROS (C) generation. ***, *P* < 0.001, compared to control. Data were mean ± SD from three independent experiments. *n* = 3 for all bar graphs.


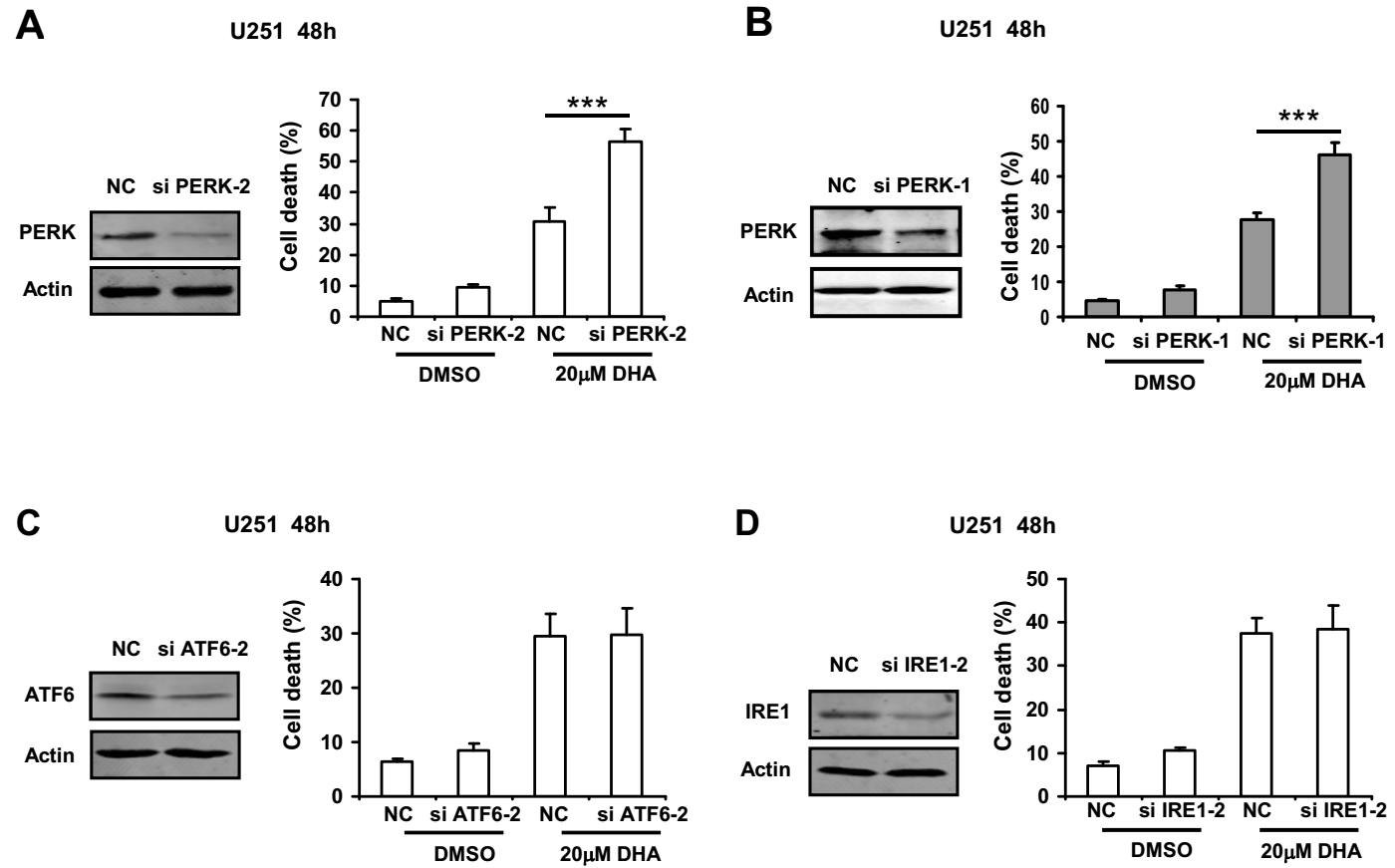


**Figure S5.** DHA-induced glioma cell death after silencing PERK (A & B), IRE1 (C) and ATF6 (D). ***, *P* < 0.001, compared to control. Data were mean ± SD from three independent experiments. *n* = 3 for all bar graphs.


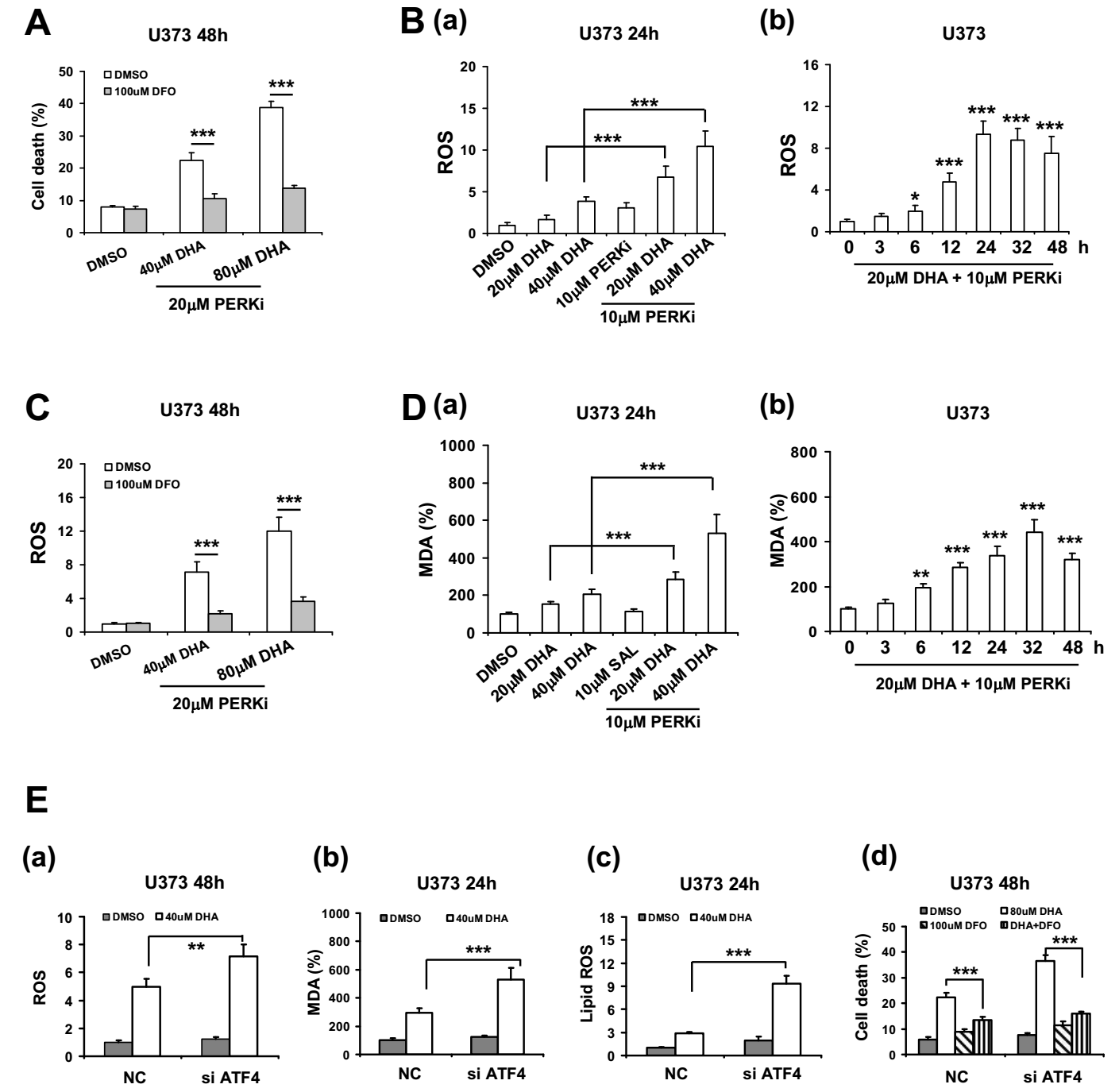


**Figure S6.** Inhibition of PERK/ATF4 signal enhanced DHA-induced ferroptosis of U373 cells. A, DFO inhibited the synergistic effects on glioma cell death of DHA combined with PERKI. B, (a) PERKI enhanced DHA-induced U373 cell ROS generation. (b) time course of ROS generation in U373 cells treated with 20 μM DHA combined with 10μM PERKI. C, DFO suppressed the synergistic effects on ROS production of DHA combined with PERKI. D, (a) PERKI enhanced DHA-induced U373 cell MDA production. (b) time course of MDA production in U373 cells treated with 20 μM DHA combined with 10μM PERKI. E, ATF4 siRNA enhanced DHA-induced ROS (a), MDA (b) and lipid ROS (c) generation in U373 cells as well as cell death (d). *, *P* < 0.05; **, *P* < 0.01; ***, *P* < 0.001. Data are mean ± SD from three independent experiments. *n* = 3 for all bar graphs.


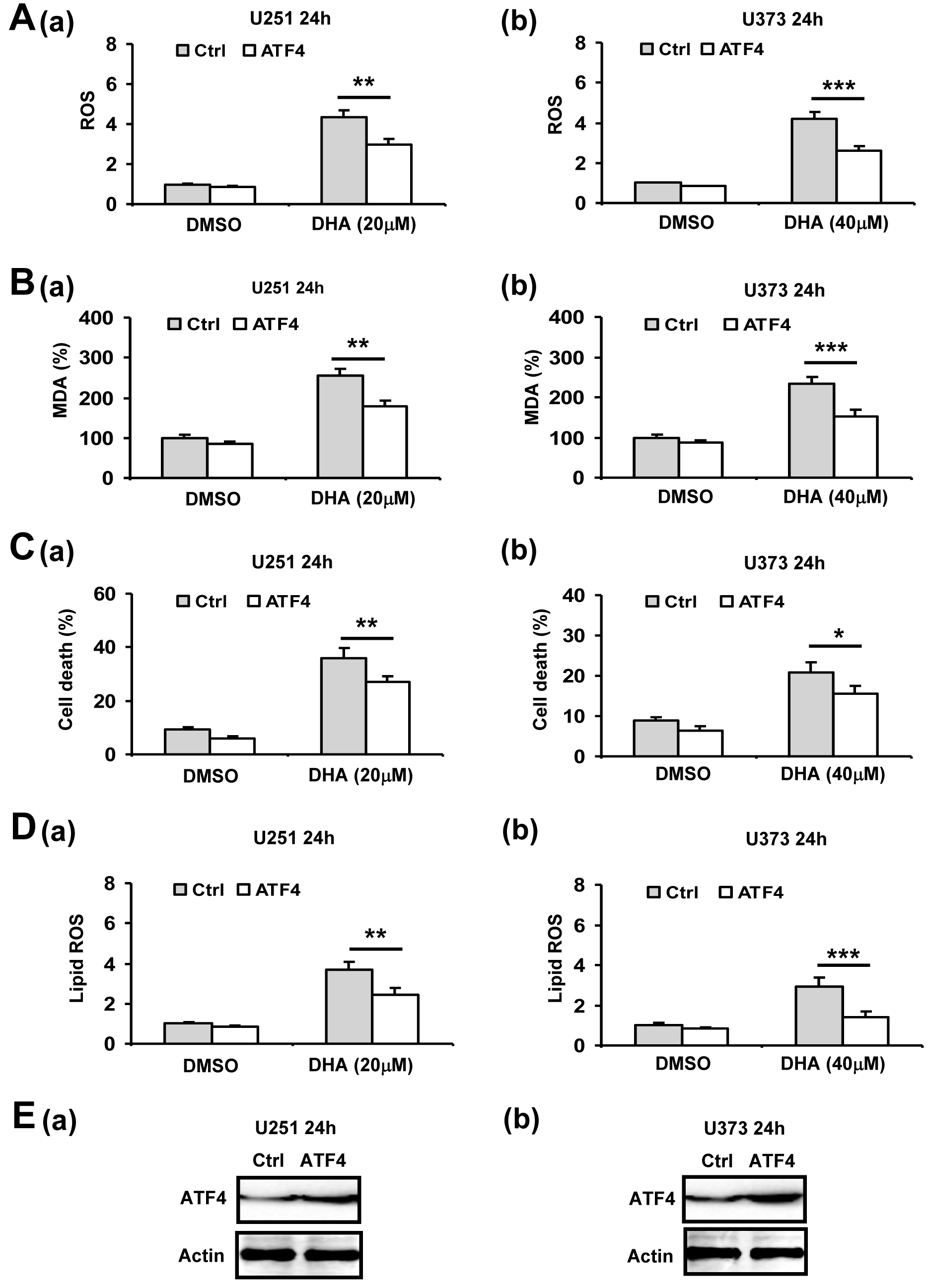


**Figure S7.** Effects of ATF4 overexpression (E) on DHA-induced ROS, lipid ROS, and MDA generation as well as cell death in glioma cells. *, *P* < 0.05; **, *P* < 0.01; ***, *P* < 0.001. Data are mean ± SD from three independent experiments.


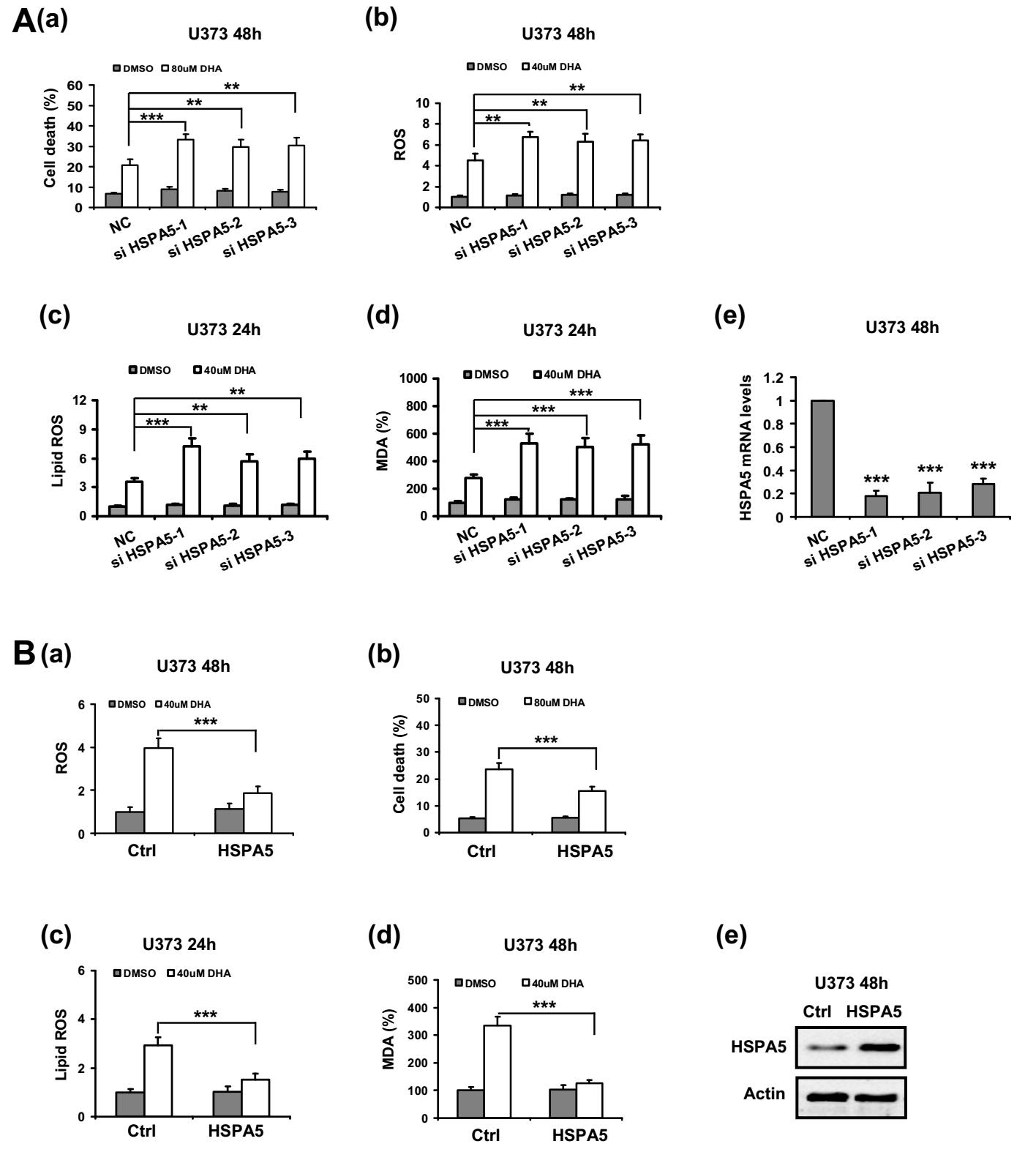


**Figure S8.** ATF4-induced HSPA5 prevented DHA-induced ferroptosis in U373 cells. A, HSPA5 silencing (e) enhanced DHA-induced glioma cell death (a), and ROS (b), lipid ROS (c) and MDA (d) generation. C, HSPA5 overexpression (e) inhibited DHA-induced glioma cell death (b), and ROS (a), lipid ROS (c) and MDA (d) generation. **, *P* < 0.01; ***, *P* < 0.001. Data are mean ± SD from three independent experiments. *n* = 3 for all bar graphs.


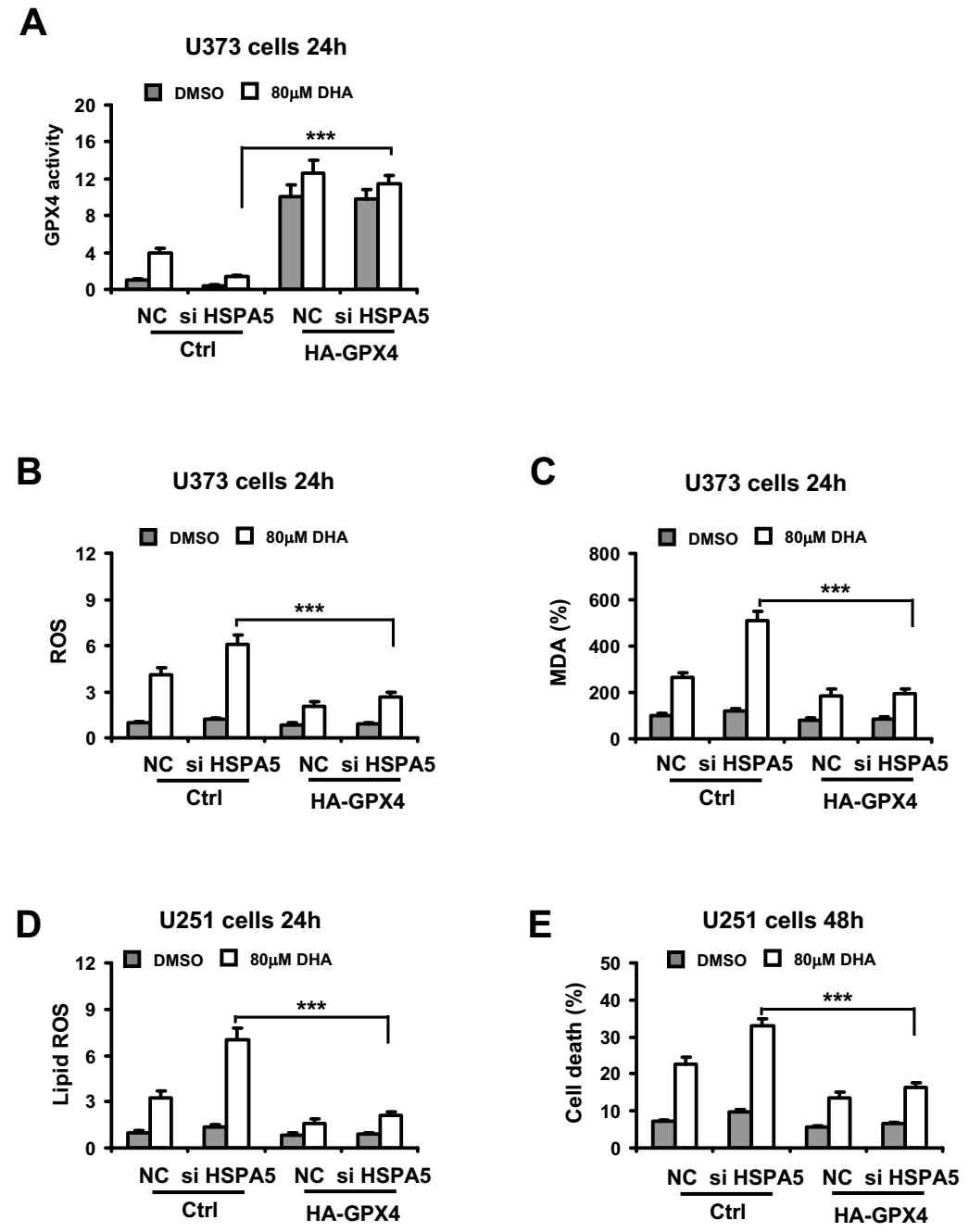


**Figure S9.** HSPA5 protected against DHA-induced ferroptosis by increasing GPX4 in U373 cells. A, GPX4 overexpression compensated HSPA5 siRNA-induced reduction of GPX4 activity in U373 cells treated with DHA. B-E, GPX4 overexpression abolished HSPA5 siRNA-induced enhancement of ROS, MDA and lipid ROS production and cell death in U373 cells treated with DHA, respectively. ***, *P* < 0.001. Data are mean ± SD from three independent experiments. *n* = 3 for all bar graphs.
